# Reduced exploratory behavior in neuronal nucleoredoxin knockout mice

**DOI:** 10.1016/j.redox.2021.102054

**Published:** 2021-06-23

**Authors:** Bao Ngoc Tran, Lucie Valek, Annett Wilken-Schmitz, Dominik Christian Fuhrmann, Dimitry Namgaladze, Ilka Wittig, Irmgard Tegeder

**Affiliations:** a Institute of Clinical Pharmacology, Goethe-University, Medical Faculty, Frankfurt, Germany; b Institute of Biochemistry I, Goethe-University, Medical Faculty, Frankfurt, Germany; c Functional Proteomics Group, Institute of Cardiovascular Physiology, Goethe-University, Medical Faculty, Frankfurt, Germany

**Keywords:** Redoxin, Calcium calmodulin kinase, Pleasure, Play, Proteomics, Behavior, IntelliCage, Camk2a, calcium calmodulin kinase 2a, NXN, nucleoredoxin, OFT, Open Field Test, EPM, Elevated Plus Maze, Nes, nestin, CoIP, coimmunoprecipitation, NO, nitric oxide, LTP, long term potentiation, Ctx, cortex, Cbl, cerebellum, HC, hippocampus, SVZ, subventricular zone, WNT, wingless, Dvl, disheveled

## Abstract

Nucleoredoxin is a thioredoxin-like redoxin that has been recognized as redox modulator of WNT signaling. Using a Yeast-2-Hybrid screen, we identified calcium calmodulin kinase 2a, Camk2a, as a prominent prey in a brain library. Camk2a is crucial for nitric oxide dependent processes of neuronal plasticity of learning and memory. Therefore, the present study assessed functions of NXN in neuronal Nestin-NXN^-/-^ deficient mice. The NXN-Camk2a interaction was confirmed by coimmunoprecipitation, and by colocalization in neuropil and dendritic spines. Functionally, Camk2a activity was reduced in NXN deficient neurons and restored with recombinant NXN. Proteomics revealed reduced oxidation in the hippocampus of Nestin-NXN^-/-^ deficient mice, including Camk2a, further synaptic and mitochondrial proteins, and was associated with a reduction of mitochondrial respiration. Nestin-NXN^-/-^ mice were healthy and behaved normally in behavioral tests of anxiety, activity and sociability. They had no cognitive deficits in touchscreen based learning & memory tasks, but omitted more trials showing a lower interest in the reward. They also engaged less in rewarding voluntary wheel running, and in exploratory behavior in IntelliCages. Accuracy was enhanced owing to the loss of exploration. The data suggested that NXN maintained the oxidative state of Camk2a and thereby its activity. In addition, it supported oxidation of other synaptic and mitochondrial proteins, and mitochondrial respiration. The loss of NXN-dependent pro-oxidative functions manifested in a loss of exploratory drive and reduced interest in reward in behaving mice.

## 1 Introduction

Nucleoredoxin (NXN) is a redoxin that resembles oxidoreductases of the thioredoxin family [1]. It was originally detected in the nucleus giving its name, but was later localized predominantly in the cytoplasm [1,2]. NXN contains three thioredoxin-like domains, the central one comprising a catalytically active WCPPC (Trp Cys Pro Pro Cys) motif that is involved in the oxidation and reduction of disulfide bonds in target proteins [1]. A recent proteomic study found a high proportion of reduced proteins in NXN-depleted neuronal cells suggesting predominant oxidase-like activity of NXN under culture conditions in these cells [3]. NXN is unique in its structure and functionally not well studied so far. It was described as a modulator of WNT-signaling via a redox dependent interaction with the WNT inhibitor, disheveled (Dvl) [4,5]. The interaction was strengthened in reducing conditions resulting in WNT/β-catenin inhibition [4]. Oppositely, NXN oxidation promoted WNT signals [4] and thereby cell fate determination [6]. Irrespective of the oxidative state, NXN retained a pool of inactive disheveled (Dvl) by preventing its ubiquitination [7], suggesting that NXN may shield other proteins against proteasomal degradation.

Proteomic studies with SH-SY5Y cells carrying a mutation of the cysteine of the catalytic site suggested broad implications of NXN in metabolism and morphogenesis [3]. An N-ethyl-N-nitrosourea (ENU) mutation screen in mice revealed a splice site mutation in NXN in mice [8], suggesting that NXN mutations may occur in the context of genetic diseases. Indeed, mutations of NXN have been associated with a recessive form of Robinow disease, which is a rare genetic disorder mainly leading to bone deformities. According to the mouse phenotype database, full NXN deletion is embryonically lethal mainly owing to cranial defects and deformities (https://www.mousephenotype.org/data/genes/MGI:109331). The phenotype suggests that NXN has unique functions which are not compensated by other redoxins, and the functions may not be limited to its redox activities. Embryonic whole mounts revealed its broad tissue distribution, particularly brain and heart, with predominant neuronal expression in septal nuclei and hippocampus in brain sections (https://www.mousephenotype.org/data/genes/MGI:109331).

To screen for further functional partners of NXN we used a yeast-2-hybrid screen and identified calcium calmodulin kinase 2a as one strong interaction partner in a brain library. Neuronal Camk2a is mostly localized at postsynaptic sites and in dendritic spines and is involved in functional and structural forms of neuronal plasticity [[9], [10], [11], [12], [13]]. Camk2a is activated upon calcium influx via NMDA-receptors. Camk2a in turn binds to the NMDA-R and maintains its open state resulting in further calcium influx and long-term potentiation [[14], [15], [16]]. In addition, Camk2a phosphorylates and thereby activates nitric oxide (NO) synthases leading to further enhancement of NO-dependent synaptic strength [[17], [18], [19]]. Camk2a itself is a target of redox modification via S-nitrosylation and further oxidation [17,20,21].

After confirmation of the Camk2-NXN interaction and motivated by the prominent localization and function of Camk2a in neurons we generated neuron-specific Nestin-Cre driven NXN deficient mice and assessed the biological implications of NXN-deficiency for in vivo behavioral readouts of activity, sociability and learning & memory and biological effects on proteome, redox-proteome and respiratory functions.

The results show that NXN-dependent oxidation is required for exploratory behavior.

## 2 Results

### 2.1 NXN expression in the mouse brain and Nestin-driven pan-neuronal deletion

Previous studies suggested that NXN is essential for brain development. In agreement with the mouse phenotype database (https://www.mousephenotype.org/data/genes/MGI:109331) we show expression of NXN in an NXN-LacZ-reporter mice in the adult mouse brain in cortex, cerebellum, hippocampus and subventricular regions and in the peripheral nervous system in sensory ganglia and nerves (Suppl. Fig. 1). NXN was also found in heart and skeletal muscle. The wide neuronal expression suggested a dominant function in the adult brain. Therefore, we generated pan-neuronal Nestin-Cre driven NXN knockout mice (Nestin-NXN^-/-^) (Suppl. Fig. 2). These mice showed 80–90% reduction of NXN expression at RNA level in various brain regions (Suppl. Fig. 2A) and about 50% reduction of NXN at protein level (Suppl. Fig. 2B and C). Residual NXN expression may arise from glial or vascular cells (not evident in histologic studies) or incomplete Cre-recombinase transformation. Owing to the partial deletion, the mice were not embryonically lethal like full NXN knockout mice, and they were healthy throughout life, allowing us to study NXN’s functions for various aspects of behavior in adult mice up to old age. Growth curves and body weight revealed a statistically lower body weight in young mice, which disappeared beyond 9–10 weeks of age (Suppl. Fig 2D). The behavioral studies were done in mice beyond youth, and not affected by weight differences.

### 2.2 NXN interacts with Camk2a and sustains Camk2a activity

**Fig. 1:**
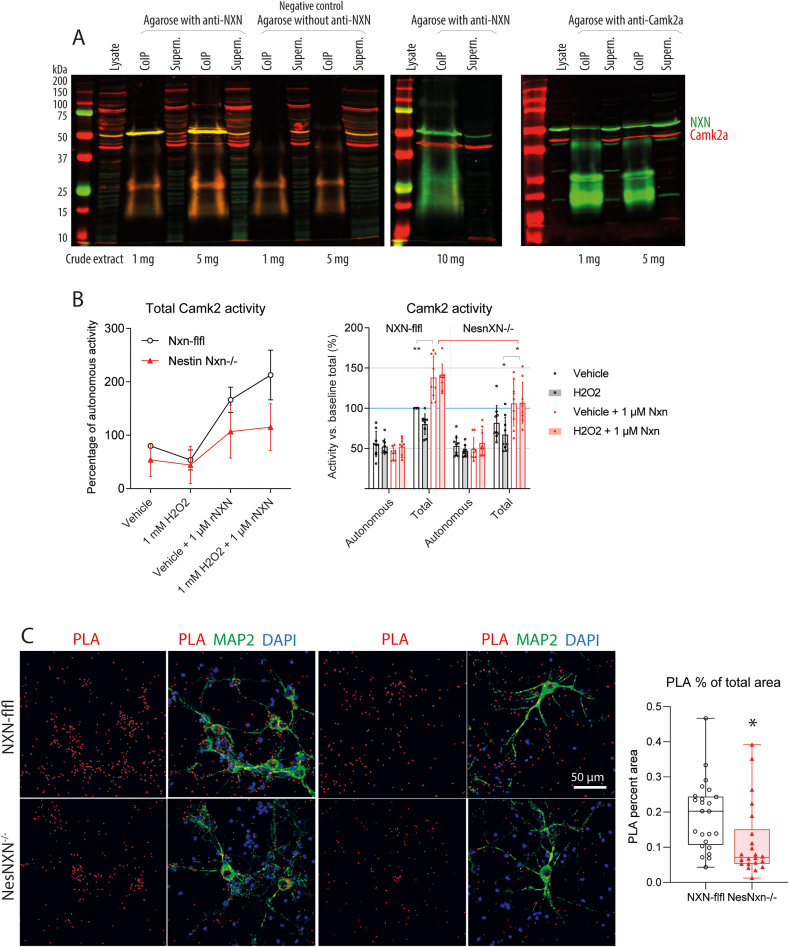
Interaction of nucleoredoxin (NXN) with calcium calmodulin kinase 2a (Camk2a). **A:** Coimmunoprecipitation of NXN and Camk2a in HEK293 cells using pull-down with anti-NXN agarose and anti-Camk2a. Western Blots were sequentially developed with secondary antibodies coupled with IR dyes and analyzed on an Odyssey system.**B:** Camk2a activity assay in Camk2-enriched mouse brain lysates of NXN-flfl and Nestin-NXN^-/-^ mice. Camk2a was enriched via pulldown with agarose beads. The “autonomous” activity is without calmodulin/without Ca^2+^, the “total” activity is with calmodulin/Ca^2+^. The tissue was obtained from three mice per genotype, each split into three samples. Data were compared per 2-way ANOVA and subsequent posthoc analysis with an adjustment of alpha according to Šidák. Asterisks denote statistical significance at *P < 0.05 (adjusted P Value). rNXN, recombinant human NXN.**C:** Proximity ligation assay (PLA, red) of NXN and Camk2a in mouse primary cortical neuron enriched cultures of each three NXN-flfl and Nestin-NXN^-/-^ mice, each split into 3 cultures. PLA signals require the proximity of the candidate proteins. The PLA dots were quantified with the “Particle counter” in ImageJ after background subtraction and default threshold setting. MAP-2 immunoreactivity (green) and DAPI (blue) were used to ensure comparable numbers of neurons. Data were compared with 2-tailed, unpaired Student’s t-test. *P < 0.05. . (For interpretation of the references to color in this figure legend, the reader is referred to the Web version of this article.)

In the first set of functional studies we screened for proteins that interact with NXN and hence might be regulated by NXN’s redox functions. A Yeast-2-Hybrid screen was performed with NXN as bait and identified two positive clones of Camk2a in a brain library (Suppl. Excel file; Short Report Suppl. Table 1). Camk2a has a number of redox-sensitive cysteines [20,21] and regulates neuronal plasticity [14,22]. There were no further convincing Y2H hits. The interaction was confirmed in both directions by coimmunoprecipitation studies in HEK293 cells using NXN-agarose beads or Camk2a beads (Fig. 1A). The co-pull down was dose dependent.

**Fig. 2:**
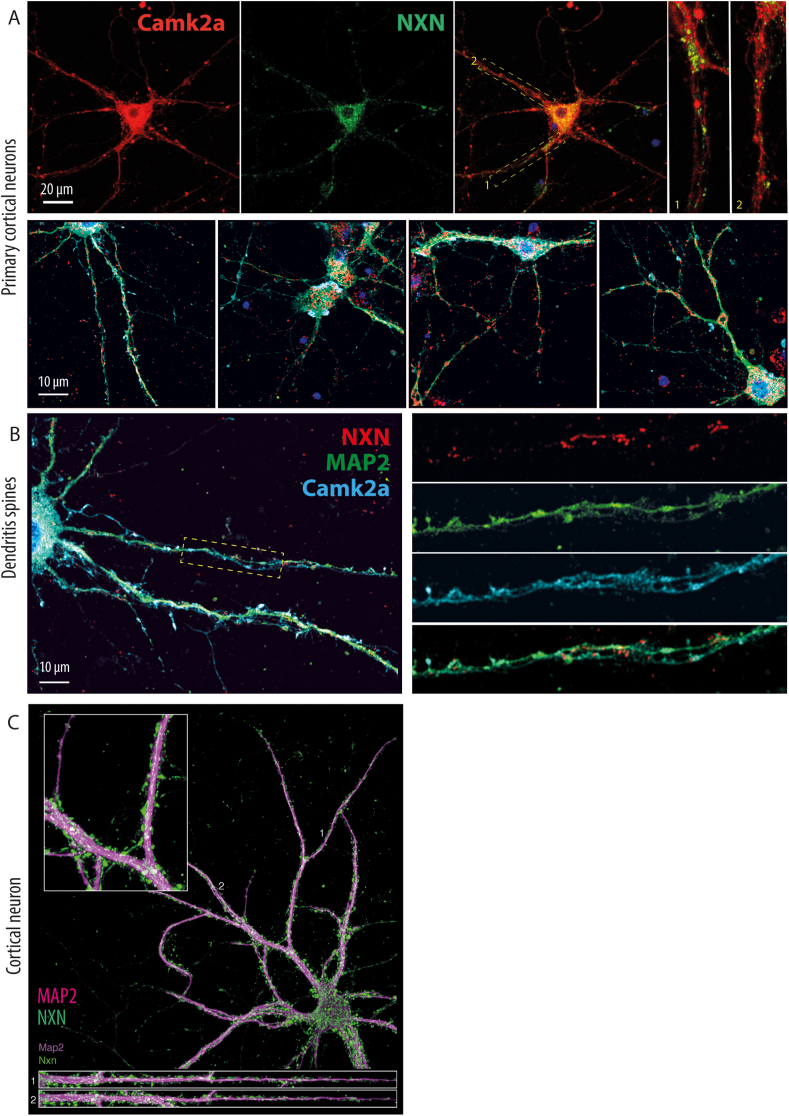
Localization and coexpression of NXN with Camk2a in primary cortical neurons **A:** Cortical neuron cultures were prepared from wildtype mice and subjected sequentially to NXN and Camk2a immunofluorescence staining. The dashed rectangles show the areas used for zoom-in of axons or dendrites.**B:** NXN localized to dendrites and dendritic spines of cortical neurons in proximity with Camk2a. **C:** Localization of NXN to dendritic spines in cortical neurons was ascertained by coimmunostaining with the axonal marker microtubule associated protein, MAP-2.

The functional implications of the NXN-Camk2a interaction were studied in a Camk2a kinase assay (Fig. 1B). Camk2a was enriched from mouse brain lysates of control mice and Nestin-NXN^-/-^ mice. There was no difference of baseline total or autonomous (without ATP/Ca^2+^) Camk2a activity between control and NXN deficient mice. H_2_O_2_ had no effect, but recombinant NXN (rNXN) significantly increased Camk2a activity. This boost was lower in samples obtained from NXN-deficient brains (Fig. 1B), suggesting a lower redox-sensitive proportion of Camk2a activity in samples of NXN-deficient mice.

For further confirmation and subcellular localization of the interaction we used a proximity ligation assay (PLA) in primary cortical neuron cultured of floxed control (NXN-flfl) and Nestin-NXN^-/-^ mice (Fig. 1C). A positive PLA signal requires that both protein candidates are in close proximity for amplification. The PLA signals mainly occurred along axons and dendrites, and the quantification revealed a significant reduction in Nestin-NXN^-/-^ mice. As expected from the Western Blot studies of the knockout mice (Suppl. Fig. 2B and C), PLA signals were not completely lost in Nestin-NXN^-/-^ neurons.

### 2.3 NXN is localized in neuronal fibers

**Fig. 3:**
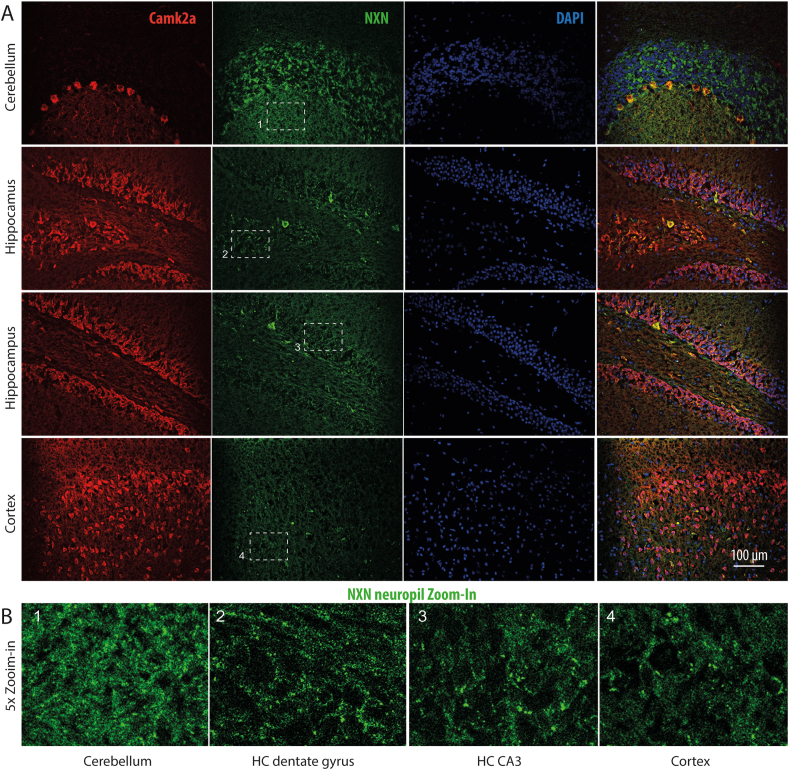
Localization and coexpression of NXN with Camk2a in brain regions A, B: Immunofluorescence analysis of NXN and Camk2a in NXN-flfl mice cerebellum, hippocampus and cortex. The dashed rectangles show the areas used for zoom-ins shown in the bottom row in panel B. NXN was mainly expressed in neuronal fibers in different brain regions.

Camk2a is expressed at postsynaptic sites in dendritic spines and crucial for NO-dependent forms of neuronal plasticity [14,[23], [24], [25]]. The PLA dots along axons suggested a similar distribution of NXN, and a putative redox modulation of spine biology. Co-immunofluorescence studies of NXN and Camk2a in primary neurons (Fig. 2A–C) or tissue (Fig. 3 A, B) revealed a neighboring expression of the candidates mainly in neuronal fibers, neurites and axons. High magnification and 3D reconstruction with co-staining of the axonal marker MAP2 (Fig. 2C) indeed suggested an expression of NXN in dendritic spines. In the cortex, hippocampus and cerebellum, there was a predominant immunoreactive signal of NXN in fibers (Fig. 3A and B).

### 2.4 Reduction of protein oxidation and mitochondrial respiration in Nestin-NXN-/- mice

**Fig. 4:**
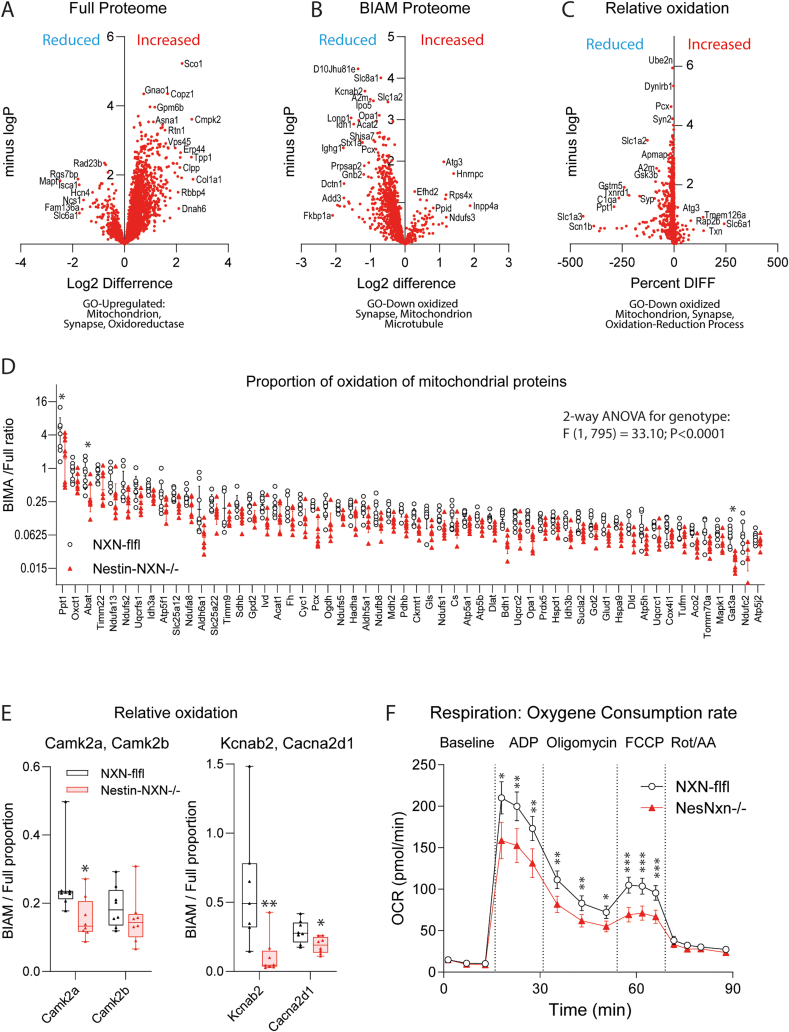
Proteomic analysis of protein expression, protein oxidation and mitochondrial respiration **A, B:** Volcano plots of protein expression (full proteome) and protein oxidation (BIAM proteome) in the hippocampus of adult Nestin-NXN^-/-^ versus NXN-flfl mice. The plots show the fold difference (=Log2 difference) on the x-axis versus the negative Log10 of the *t*-test P-value. Candidates are labeled. Proteins on the left side of the Y-axis were reduced in Nestin-NXN-/- mice, proteins on the right were increased.**C:** Volcano plots of relative protein oxidation (BIAM/Full proteome in percent). The x-axis shows the differences between the percent oxidation of Nestin-NXN^-/-^ versus NXN-flfl mice.**D:** Scatter plots of the proportion of protein oxidation of proteins associated with the GO-term "mitochondrion", a key regulated pathway. The proportion of protein oxidation was reduced in Nestin-NXN^-/-^ mice (2-way ANOVA). The asterisks show significant differences of individual proteins, *P <0.05 adjusted according to Benjamini-Hochberg FDR.**E:** Proportion of protein oxidation of Camk2a, Camk2b and the voltage gated ion channels, Kcnab2 (potassium) and Cacna2d1 (calcium). The box is the interquartile range, the line is the median, the scatter shows individual mice and the whiskers minimum to maximum. *t-test P < 0.05.**F:** Mitochondrial respiration in a Seahorse analyser. The “substrate-uncoupler-inhibitor-titration” protocol measured basal respiration, subsequently coupled respiration by adding ADP substrate, followed by oligomycin to assess ATP generation and proton leakage, subsequently FCCP to assess maximum uncoupled respiration, and finally rotenone (Complex-I inhibitor) plus antimycin A (Complex-III inhibitor) to asses residual non-mitochondrial respiration. Oxygen consumption (OCR, mean ± 95% CI) was analyzed with Wave® software. The analysis was done with n = 96 NXN-flfl samples and n = 69 Nestin-NXN^-/-^ samples of mitochondrial fractions of each three mouse brains. Time courses were compared by 2-way ANOVA for "group" X "stimulus" and posthoc analysis using an adjustment of alpha according to Šidák. Asterisks reveals significant differences between groups. *P<0.05, **P<0.01, ***P<0.001.

To assess the functional implications of NXN in neuronal fibers we analyzed the full proteome and oxidized proteome in the hippocampus of Nestin-NXN^-/-^ and NXN-flfl mice. Scatter plots of the full proteome (Suppl. Fig. 3A) and the BIAM redoxome (Suppl. Fig. 3B) show a linear even distribution of proteins and oxidized proteins in Nestin-NXN^-/-^ and NXN-flfl mice. Volcano plots (Fig. 4A and B) revealed that protein oxidation (BIAM, Fig. 4B) was reduced in Nestin-NXN^-/-^ mice, which was also evident in analysis of relative protein oxidation (BIAM) versus full protein expression (Fig. 4C), including only proteins, which were detected in both proteomes. The data suggested that NXN mainly acted as an oxidase in agreement with a previous study in neuronal cells [3], so that more proteins were in a reduced state in Nestin-NXN^-/-^ mice. Gene Ontology analyses for down-oxidized proteins (P value < 0.1) revealed an association with the GO terms "mitochondrion" (Fig. 4D), "synapse" (Suppl. Fig. 3C) or oxidoreductase processes" (Suppl. Fig. 3D), and Camk2a was significantly down-oxidized (Fig. 4E). Further candidate down-oxidized proteins at the synapse were synaptic vesicle proteins (synaptoporin, synaptophysin), glutamate decarboxylase 1 (Gad1) and the AMPA receptor subunit Gria1 (Suppl. Fig 3D) and the voltage gated ion channels subunits, Kcnab2 (potassium) and Cacna2d1 (L-type calcium) (Fig. 4E). The mass spectrometry proteomics data have been deposited to the ProteomeXchange Consortium via the PRIDE [26] partner repository with the dataset identifier PXD024624.

A lowering of oxidation of mitochondrial proteins suggested differences in mitochondrial respiration. Hence, we measured respiratory oxygen consumption using a “substrate-uncoupler-inhibitor-titration” protocol in mitochondria isolated from brain tissue of Nestin-NXN^-/-^ and NXN-flfl mice (Fig. 4F). Mitochondria were subjected to a Seahorse SUIT analysis. Baseline respiration was equal in both groups, but oxygen consumption was reduced in mitochondria of Nestin-NXN^-/-^ brains after adding ADP substrate to measure ATP-coupled respiration. A lower rate of oxygen consumption was maintained after adding the uncoupler oligomycin to measure the proton leak, and after FCCP to measure peak uncoupled respiration. Residual non-mitochondrial respiration, after adding rotenone and antimycin A to block complex I and III, did not differ between genotypes. The data suggested a reduction of the respiratory rate in NXN-deficient neurons, possibly leading to a lowering of ATP generation.

Nestin-NXN^-/-^ mice show reduced exploratory behavior and low interest in the reward.

To assess the in vivo implications of neuronal NXN deficiency, Nestin-NXN^-/-^ versus NXN-flfl mice were subjected to complex behavioral analyses. Camk2a mediated plasticity and spine remodeling are crucial for learning and memory, social behavior and exploratory drive [[27], [28], [29]]. We used IntelliCages, touchscreen chambers and mazes to assess these aspects of cognition.

**Fig. 5:**
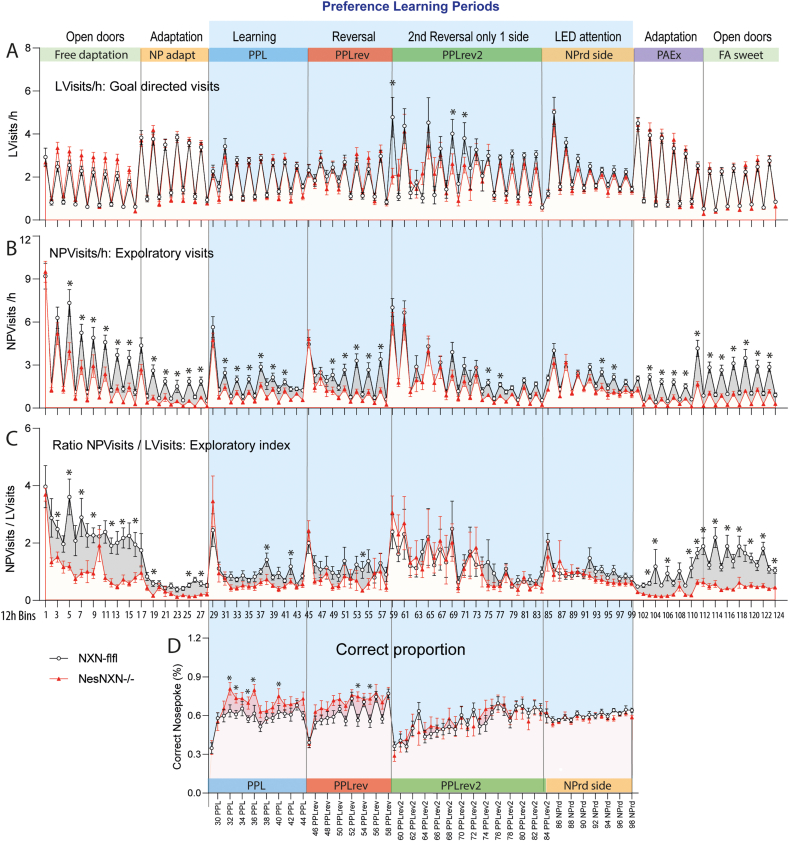
Exploratory and goal directed activity of Nestin-NXN^-/-^ versus NXN-flfl mice in IntelliCages, **A:** Time course of corner visits per hour (Visits/h) during different tasks in IntelliCages.**B:** Time course of exploratory visits with nosepokes but without licks (NPVisits/h).**C:** Time course of the ratio of exploratory visits (NPVisits, with nosepokes but without licks) versus goal directed visits (LVisits, visits with licks). **D:** Proportion of correct corner visits during place preference learning tasks The tasks are shown in the header and described in Suppl. Table 6. The data show means ± sem of 14–15 mice per group. The fluctuation of the behavior reveals nighttime and daytime differences (12h Bins). Data were compared with 2-way ANOVA for the factors “time” X “genotype” and posthoc comparison for “genotype” with adjustment of alpha according to Šidák. Abbreviations: FA, free adaptation; NP, nosepoke; PPL, place preference learning; PPLrev, place preference reversal learning; NPrd, random assignment of correct side with LED; PAEx, place avoidance extinction.

**Fig. 6:**
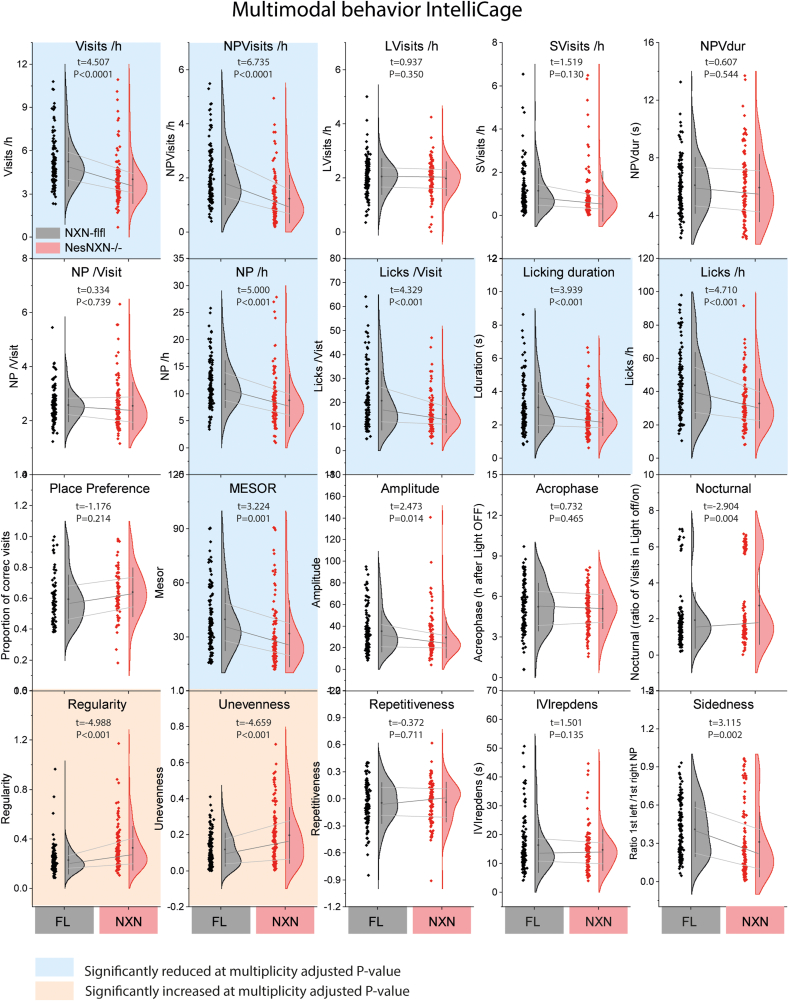
IntelliCage behavior of Nestin-NXN^-/-^ versus NXN-flfl mice averaged over different tasks To compare behavior across modules/tasks the 12h cycle averages for each mouse per period were pooled and were plotted as half violin plots that show the Gauss distribution. The box within the violin is the interquartile range, the median is the dot. The lines connect the mean and the quartiles. The left scatters show individual results, each mouse is represented by eight dots for each of the eight modules/tasks. The data were compared per 2-tailed, unpaired Student’s t-test for each parameter. The parameters are explained in Suppl. Table 7. Asterisks show statistically significant differences between genotype. *P< 0.05; **P<0.01; ***P<0.001.

**Fig. 7:**
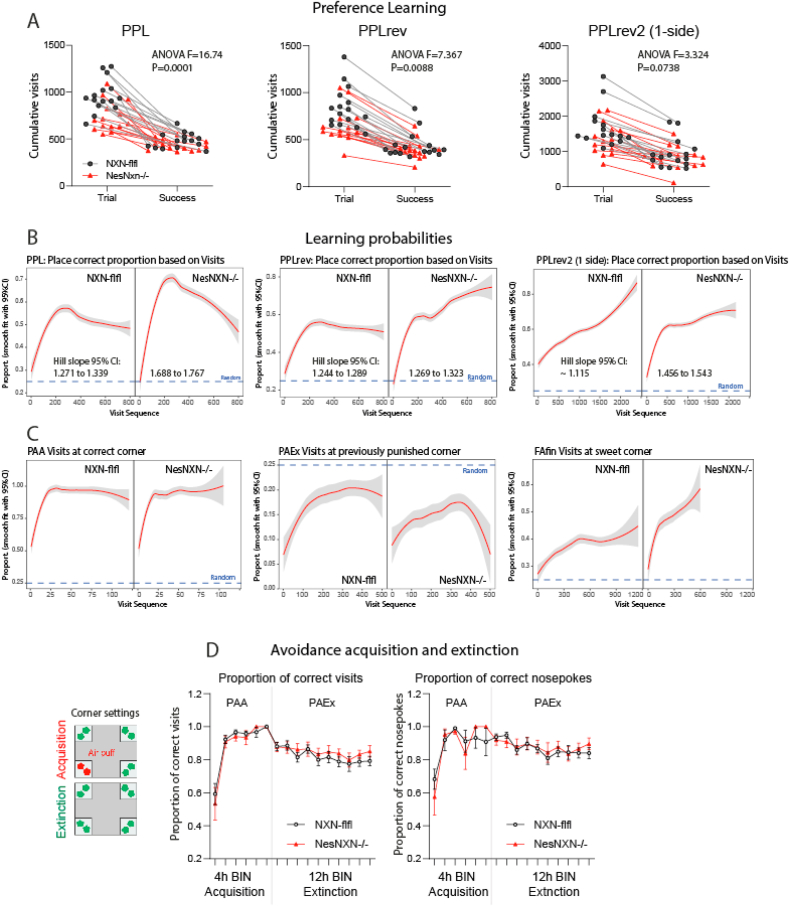
Learning and memory of Nestin-NXN^-/-^ versus NXN-flfl mice in IntelliCages **A:** Paired scatter/line plots show the numbers of trials versus successes for place preference learning and reversal learning tasks in IntelliCages. The groups comprised 14–15 mice per genotype. Each scatter shows a mouse. Data were compared with 2-way ANOVA for “trial/success” versus “genotype and reveal a lower number of trials of Nestin-NXN^-/-^ mice but similar successes. Abbreviations: PPL, place preference learning; PPLrev and PPLrev2, PPL reversal learning and second reversal learning. B**:** Learning probability curves show the proportion of correct corner visits relative to the trial number during place preference learning and PPL reversal learning (PPLrev, PPLrev2) tasks. The learning probability curves were significantly steeper during PPL in Nestin-NXN^-/-^ as revealed by non-overlapping 95% confidence levels for the Hill factor (Hill factor 95% CI given in the figures). Nestin-NXN^-/-^ needed fewer trials to reach 35% accuracy which was defined as learning threshold (10% above random success).**C:** In analogy to B, the learning probability curves show the proportion of correct corner visits during place avoidance acquisition (PAA), place avoidance extinction (PAEx) and final free adaptation with one corner providing sweet water. In PAEx, all corners were allowed, only an LED reminded of the previously forbidden/punished corner. The proportion of correct corners in this task reveals maintenance of memory for the previously punished corner. The steepness of the curves did not differ significantly between genotypes.**D:** Time courses of the proportion of correct visits and correct nosepokes during place avoidance acquisition (PAA) and place avoidance extinction (PAEx). The left image shows the task settings. In PAA, a nosepoke in the forbidden corner triggers an airpuff. LED is used as support of memory. In PAEx, all corners are free, but the LED reminds of the previously punished corner. The data show means ± sem of 14–15 mice per group. There were no differences between genotypes.

Nestin-NXN^-/-^ and NXN-flfl mice were continuously observed in IntelliCages over nine weeks starting with free adaptation and proceeding to complex learning and reversal learning tasks (Fig. 5, Fig. 6, Fig. 7). Visits, nosepokes and licks were the primary readouts (Fig. 5, Suppl. Fig. 4), which allowed for a detailed description of multiple components of the behavior (Fig. 6).

The overall activity as revealed by the total number of visits per hour (Visits/h) (Fig. 5A) and the ratio of nosepokes per visit with nosepokes (NP/NPVisit) (Suppl. Fig 4A) did not differ between genotypes throughout the experiments. Daytime to nighttime fluctuations were similar and regular in both genotypes suggesting overall cognitive health of Nestin-NXN^-/-^ mice. For comparison, alterations of visits, circadian fluctuations and of the NP/NPVisit ratio occur in models of dementia [30] or traumatic brain injury [31].

However, the numbers of non-goal directed visits, in which mice make nosepokes but do not lick (NPVisits/h) was reduced in Nestin-NXN^-/-^ mice throughout the observation, most strongly during non-learning periods where NPs are made for exploration rather than to get reward (Fig. 5B). Accordingly, the ratio of exploratory visits (= NPVisits) versus goal-directed visits (= visits with licks, LVisits) was strongly reduced (Fig. 5C). This ratio provides an exploratory index. The differences between genotypes were stronger during free adaptive, non-learning periods.

During the initial easy place preference learning periods, Nestin-NXN^-/-^ mice showed a higher accuracy (proportion of correct nosepokes) likely owing to low exploration (Fig. 5D). The advantage in terms of accuracy was lost during the more difficult place preference learning periods. Overall, Nestin-NXN^-/-^ mice were less interested in the reward. Licking during the successful LVisits was reduced throughout the observation in terms of the numbers and the licking duration (Suppl. Fig 4, Fig 6). Hence, Nestin-NXN^-/-^ mice mostly restricted licking to the need, but like the controls, they increased licking in the final free adaptation protocols in which sweet water was offered (Suppl. Fig. 4B).

To reveal the behavioral pattern, twenty behavioral parameters were summarized over the full time course and presented as violin plots (Fig. 6). The violin plots show multiplicity adjusted statistics highlighted in red (significantly increased) or blue (significantly reduced) and reveal that the major difference between genotypes are a reduction of exploratory, non-goal-directed NPVisits, overall visiting activity and licking, and an increase of Regularity and Unevenness, which both show strong habits. High “Regularity” indicates a low frequency of diagonal transitions. High “Unevenness” shows strong spontaneous usage of specific corners instead of equal usage of all four corners. It is an indicator of strong habits and agrees with a low exploratory drive.

### 2.5 Learning and memory in Nestin-NXN^-/-^ mice

The proportion of correct visits (Fig. 5D) suggested that low-exploratory behavior of Nestin-NXN^-/-^ was associated with a learning advantage. Therefore, further cognitive IntelliCage parameters of preference and avoidance learning and touchscreen behaviors were studied.

Indeed, during the initial easy preference learning (PPL) tasks in the IntelliCage, Nestin-NXN^-/-^ needed fewer trials to reach the criterion of success, which was set at 35% correct responses, i.e. 10% above random (Fig. 7A). In agreement, the learning probability curves were significantly steeper during the easy PPL task (Fig. 7B). There was no difference in the difficult Reversal PPL tasks (Fig. 7B). There was also no difference for place avoidance acquisition or extinction (Fig. 7C), or in the final free trial which assessed the appeal or addiction to sweet (Fig. 7C, right). The proportion of correct nosepokes and correct visits during avoidance learning and extinction was also alike (Fig. 7D). We infer that Nestin-NXN^-/-^ behave goal-directed and have strong habits, but they are less curious or “adventurous”, leading to a mild advantage in easy preference learning but not in Reversal learning or avoidance learning and memory. The most remarkable behavioral trait is low exploration.

**Fig. 8:**
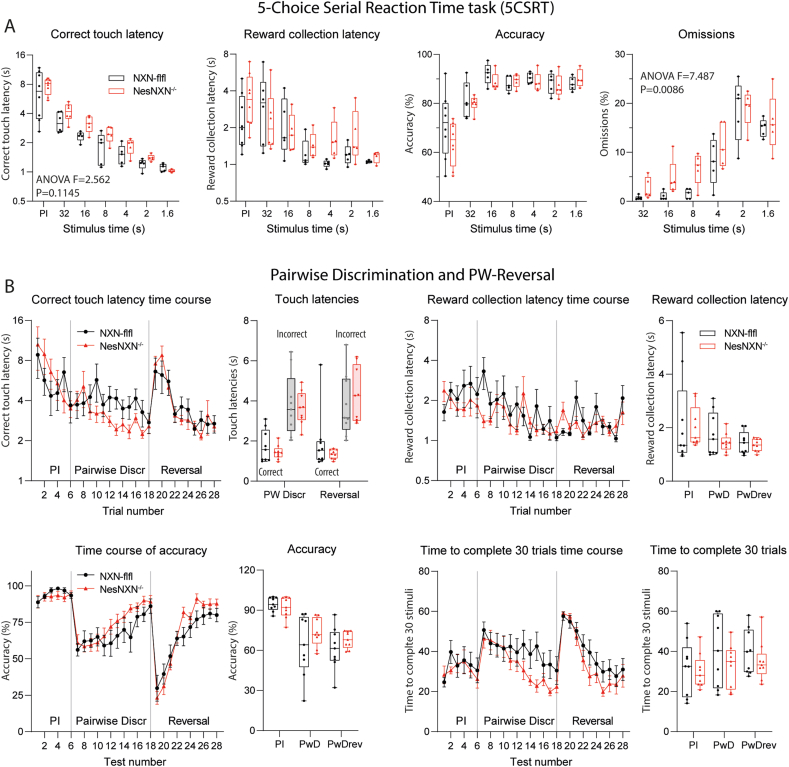
Attention and discrimination learning of Nestin-NXN^-/-^ versus NXN-flfl mice in touchscreen task **A:** Correct touch latency, reward collection latency, accuracy and percentage of omissions in a 5-choice serial reaction time (5CSRT) touchscreen task as described in Suppl. Table 5. A white square is enlightened randomly at one out of 5 possible locations on the screen with decreasing stimulus duration. PI, “punish incorrect” training. The mouse gets reward for touching the enlightened square. The tests measure attention and response velocity. The data show means ± sem of 8 mice per genotype. They were compared with 2-way ANOVA for “stimulus time” X “genotype” and posthoc analysis according to Šidák. Nestin-NXN^-/-^ mice show a higher percentage of omissions (stimuli without response).**B:** Correct touch latency, reward collection latency, accuracy and time needed to complete 30 trials in a pairwise discrimination and reversal (PwD, PwDrev) touchscreen task (Suppl. Table 5). The data show means ± sem of 8 mice per genotype. The test was done after the 5CSRT task with the same groups of mice. Two images are presented on the screen. The mouse has to touch the correct image. After learning, the denotation of correct is reversed. It is a test of discrimination learning and reversal learning. Data were compared with 2-way ANOVA for “stimulus time” X “genotype”. There were no differences between genotypes.

Nestin-NXN^-/-^ versus NXN-flfl mice were next observed in touchscreen learning chambers (Fig. 8A). In the 5-choice serial reaction time task (5CSRT), which measures attention and response velocity, Nestin-NXN^-/-^ were somewhat slower in terms of reward collection (n.s.) and made more omissions when the stimulus time was short. The behavior agrees with lower attention to objects, here the enlightened rectangle on the screen. There were no differences in the Paired Discrimination touchscreen test (Fig. 8B), which requires discrimination and learning of visual objects and is sensitive to cholinergic signaling [32,33]. Neither accuracy nor latencies for screen touch or reward collection differed between genotypes.

### 2.6 Lower interests in novel objects or environments and low voluntary wheel running

**Fig. 9:**
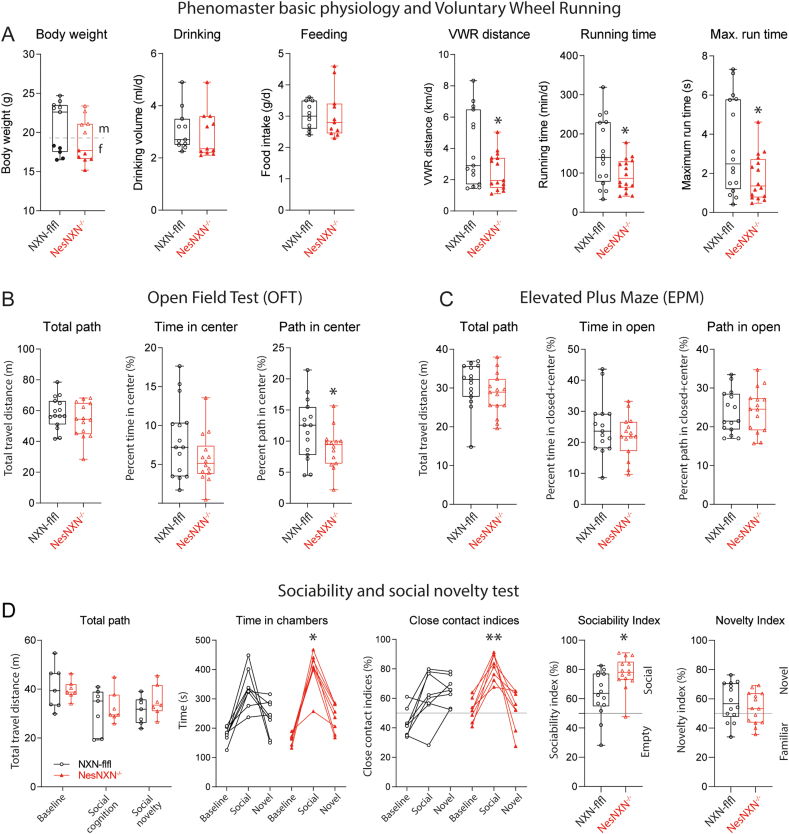
Voluntary wheel running and Maze tests of Nestin-NXN^-/-^ versus NXN-flfl mice **A:** Body weights, drinking, feeding and voluntary wheel running (VWR) of NXN-flfl and Nestin-NXN-/- mice in Phenomaster Cages, which provide a home environment with PC-controlled running wheel and precision scales for food and water bottle. Data show the means ± sem of 11 mice per genotype, and were compared with 2-tailed, unpaired Student’s t-tests. The asterisk denote statistical significance, *P < 0.05.**B:** Open field behavior of NXN-flfl and Nestin-NXN^-/-^ mice. The total path shows the overall activity. Time and path in the center compartment reveal the exploratory behavior. Data show the means ± sem of 14–15 mice per genotype, and were compared with 2-tailed, unpaired Student’s t-tests, *P < 0.05.**C:** Elevated Plus Maze behavior of NXN-flfl and Nestin-NXN^-/-^ mice. The total path shows the overall activity, time and path in the open arms reveal the exploratory behavior. Data show the means ± sem of 14–15 mice per genotype, and were compared with 2-tailed, unpaired Student’s t-tests. No difference between genotypes. **D:** Behavior of NXN-flfl and Nestin-NXN^-/-^ mice in a sociability and social novelty/memory three-chamber maze test. Data show the means ± sem of 7 mice per genotype. The total path shows the overall activity. Time in the social chamber and the relative time in close contact with the social compartment reveal the social interest relative to the interest in an object. Nestin-NXN^-/-^ mice had a stronger preference of social versus empty compartment. Data were compared with 2-way ANOVA and subsequent posthoc test according to Šidák (three time points) or by 2-tailed, unpaired Student’s t-tests. *P < 0.05.

The IntelliCage data suggested that Nestin-NXN^-/-^ mice were less interested in objects or environment and had a low exploratory and rewarding behavior. To further dissect the behavioral dimensions which were affected we used Maze tests and rewarding voluntary wheel running (VWR). There were no differences in feeding and drinking (Fig. 9A), but VWR was reduced in Nestin-NXN^-/-^ mice (Fig. 9A right) again suggesting a low rewarding exploratory and playful behavior. We did not observe differences of RotaRod running (not shown), where mice are forced to run.

In the Open Field Test (OFT), Nestin-NXN^-/-^ mice explored the center of the field less than the controls as revealed by shorter paths in the center compartment (Fig. 9B). Overall paths were equivalent again showing that Nestin-NXN^-/-^ mice had no motor deficit. Similarly, total paths were equivalent in the Elevated Plus Maze (EPM) (Fig. 9C) test of anxiety and in three-chamber tests of social cognition and memory (Fig. 9D). The EPM did not show any differences. Hence, low exploratory behavior of Nestin-NXN^-/-^ was not caused by anxiety.

The sociability test showed that Nestin-NXN^-/-^ had a stronger relative interest in social partners over the empty compartment. Hence, social interest was rather increased, and mice had a similar preference of familiar versus novel social partners as the NXN-flfl mice (Fig. 9D). The social behavior shows that Nestin-NXN^-/-^ mice do not show any autism-like features like mice with alterations of dendritic spine plasticity and remodeling [34,35]. It is of note, that a loss-of-function mutation of Camk2a leads to autism like behavior in mice [9], which was not the case in Nestin-NXN^-/-^ mice, and agrees with the hypothesis that NXN is a modulator but not essential.

### 2.7 Multimodal behavior reveals low exploratory drive and strong habits

**Fig. 10:**
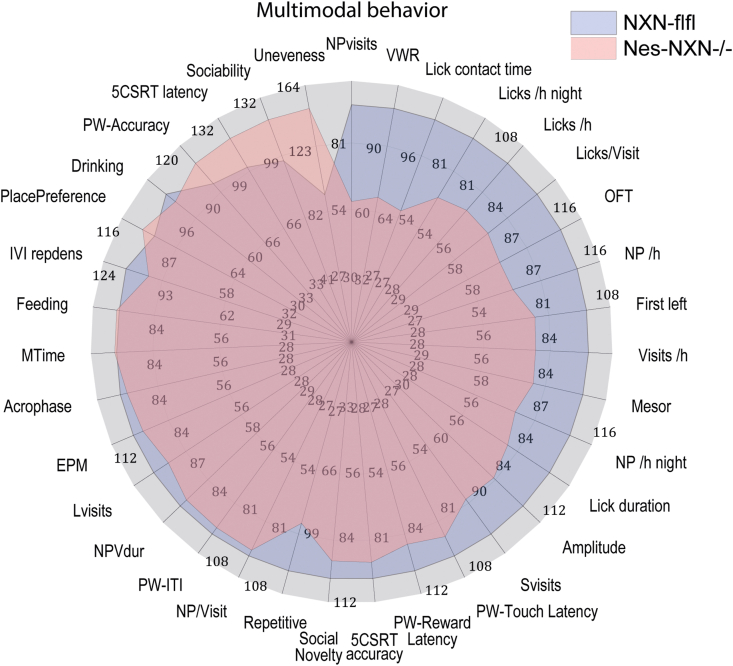
Polar plots of the behavioral pattern. Behavioral data were normalized on the median of the NXN-flfl control mice to assess the overall pattern of the behavioral phenotype as polar plots. The parameters were sorted clockwise starting with the parameter which was most reduced (NPVisits at 12 a clock). The colored behavior-areas reveal low exploration (NPVisits) and low reward providing activity (VWR) but very strong habits (Unevenness) in Nestin-NXN^-/-^ mice.

To reveal behavioral patterns across different tests to describe the affected behavioral dimensions, behavioral readouts were normalized to the median behavior of NXN-flfl control mice as percentages and displayed as Polar Plots (Fig. 10). The behavioral parameters were sorted clockwise and revealed the low exploratory behavior (NPVisits, VWR, Licking, OFT) and increase of habits (unevenness, regularity) and relative higher relative interest in social partners than for objects (sociability). Decision trees using behavioral parameters of all tests as input revealed that the genotypes could be separated according to the exploratory activity (low in NesNXN^-/-^) and regularity (high in NesNXN^-/-^) (Suppl. Fig. 5). Hence, low exploration is the primary phenotype of neuronal NXN deficiency.

## 3 Discussion

We show in the present study that the oxidoreductase, NXN, interacts with and sustains the activity of Camk2a, a postsynaptic kinase which is crucial for neuronal plasticity [[14], [15], [16]]. Camk2a has been implicated in a number of psychiatric diseases [27] including autism [9], schizophrenia [36] and addiction [[37], [38], [39]]. It is activated by calcium upon activation of NMDA receptors, is then transferred to the receptor and maintains its open state, allowing more calcium to enter and resulting in long-term potentiation [10,40]. In comparison with serious phenotypes of loss-of-function models of Camk2a leading to autisms-like behavior [9] and defects of learning and memory [12,16,41], partial deletion of NXN in neurons had subtle effects on mouse behavior, suggesting that it acted as a non-essential modulator of Camk2a.

We did not achieve complete NXN protein deletion with the Nestin-Cre driver at the protein level whereas NXN mRNA was reduced by 80–90% as expected [42], suggesting compensatory mechanisms. The NXN antibody was specific in vitro in knockdown and overexpressing cells [43], suggesting that the knockout in mice was indeed partial. This is a limitation of our studies. However, the incomplete deletion likely rescued the mice from embryonic death that occurs in non-specific pan-deletion models, which are lethal (https://www.mousephenotype.org/data/genes/MGI:109331). Hence, we did not achieve the expected near-complete deletion, but the mice allowed us to study NXN-dependent behavior in adult mice.

The predominant feature was a reduction of non-goal-directed behavior whereas goal-directed activity and learning were normal. The accuracy was even higher in easy learning tasks resulting from reduced try-and-error behavior. High motivation in mice comes at the expense of accuracy [44]. The phenotypes were revealed by multimodal analyses of mouse behavior in IntelliCages, Touchscreens and video-based observations in classical Maze tests, which all provide unbiased behavioral observations. IntelliCage observations have the advantage to study groups of mice in enriched home cages without observer handling, and provide complex behavioral patterns [45]. Based on the multimodal assessment we conclude that Nestin-NXN^-/-^ mice restricted their efforts to goal-directed behavior with lower interest in adventurous, exploratory and rewarding activities including voluntary wheel running [46,47].

Exploration in mice is a rewarding playful behavior particularly in young mice [48,49], depends on Camk2a [50], and it decreases continuously upon healthy aging in mice [44,51] likely to save energy for required activity. Hence, Nestin-NXN^-/-^ mice behaved “wise” without evidence of premature aging. While this is efficacious for laboratory mice, it may be associated with a reduction of pleasure. It is of note, that Camk2a mutations in mouse addiction models impair dopamine and serotonin mediated reward signaling and lead to earlier transition to severe cocaine use [37] or alcohol addiction [38,52]. Hence, NXN may sustain motivation and pleasure on exploration, possibly by maintaining Camk2a oxidation and activity. However, NXN may have further target proteins, which is suggested by the redox-proteomic screen in which we found a reduction of oxidation of several proteins involved in mitochondrial and synaptic function. The BIAM proteome suggests that NXN mainly acts as an oxidase which agrees with a previous study in neuroblastoma cells [3], in which the redox proteome of NXN-mutant "loss-of-function" cells was shifted to reduction [3]. Based on our results, we hypothesize that NXN promotes oxidation of redox-sensitive cysteines of Camk2a enhancing thereby its activity [17], possibly by strengthening autophosphorylation [16,53]. The most interesting cysteines in this regards are Cys280 and Cys289 (both conserved in mice and humans) [17] because they are close to the threonine autophosphorylation site T286 [54], which is crucial for exploratory behavior [55], learning [14,16] and addiction [38,52]. One may speculate that modulation of the redox status alters phosphorylation and thereby function [56]. The BIAM redox proteome screen detected a carbamylation site cysteine (Cys373) and methionine oxidation sites in close vicinity, whose functions are unknown. Irrespective of Camk2a, the observed low oxidation state in Nestin-NXN^-/-^ brains suggest that NXN-dependent pro-oxidation promotes mitochondrial respiration and that the energy is needed for a youthful exploratory drive at the behavioral level.

In summary, we show that partial loss of NXN in neurons leads to a reduction of exploratory behavior with normal learning and sociability. The interaction with Camk2a and loss of Camk2a oxidation upon NXN deletion suggests that the behavioral manifestations involve redox modification of Camk2a, which are contributed by further NXN-dependent protein oxidations of synaptic and mitochondrial proteins. The NXN knockout phenotype was not a mirror of Camk2a gain or loss models [9,27,57] but would agree with a subtle modulation of Camk2a. Translated to humans, NXN inhibition might reduce hyper-motivation and hyperactivity in favor of goal-directed activity and calmness, but possibly at the expense of adventure or discovery.

## 4 Methods

### 4.1 Yeast-2-hybrid

The Y2H screen was done for human fetal brain and bone marrow libraries with the Matchmaker GAL4 system (Clontech) using Gateway Invitrogen plasmids for nucleoredoxin as bait according to standard protocols [58]. Preys were categorized according to established selection criteria [59]. True positives were detected more than once, started in the coding region and had low promiscuity scores. Results were considered as false positive if transcription started in 3' UTR resulting in unnatural peptides, if transcription started far in 5' UTR (>50 bases), if the prey was found with several unrelated baits according to the database (promiscuity score ≥ 4), and if preys were detected only once unless the prey had a biological relation to NXN.

### 4.2 Mice

Floxed nucleoredoxin (NXN) mice were obtained from the European Conditional Mouse Mutagenesis Program (EUCOMM; Nxn^tm1a^(EUCOMM)^Wtsi/Ieg^). The mice carry a conditional-ready allele, which was inserted by homologous recombination. In the construct, exon 2 of mouse NXN (ENSMUSG00000020844) is flanked with loxP sites for Cre mediated deletion. In front is a promoter-less FRT-flanked LacZ-Neo reporter cassette, so that LacZ is expressed under the control of the NXN promotor and were used to assess NXN expression and localization. The reporter was then removed by breeding with Flp-mice and subsequently, Nxn-flfl were crossed with Nestin-Cre mice (The Jackson Laboratory, B6.Cg-Tg(Nes-cre)^1Kln/J^) to cut out exon 2 and create a pan-neuronal Nestin-NXN^-/-^ knockout mouse. Reportedly, Cre recombinase activity is present by embryonic day 11. Nestin-Nxn^-/-^ have a C57BL6N genetic background. The genotyping followed the protocol provided by EUCOMM. The successful pan-neuronal deletion was confirmed at RNA and protein level.

Experiments were performed with Nestin-NXN^-/-^ and Nxn-flfl litter mates. The sample sizes depended on the experiments and readouts. Groups comprised 5–16 mice per genotype at 8 to 40 weeks of age as specified in Suppl. Table 2 and the figure legends. Mice were allowed to acclimatize to the experiment rooms, cages or mazes before starting experiments. They had free access to food and water and were maintained in climate-controlled rooms at a 12 h light-dark cycle.

The experiments were approved by the local Ethics Committee for Animal Research (Darmstadt, Germany) and adhered to the European guidelines and to those of GV-SOLAS for animal welfare in science and agreed with the ARRIVE guidelines.

### 4.3 RNA extraction and RT-PCR

Total RNA was extracted from homogenized cells or tissue with Trizol reagent and reverse transcribed using Oligo-dT or random hexamers as a primer to obtain cDNA fragments. QRT-PCR was performed using a QuantStudio 5 Real-Time-PCR-System (ThermoFisher) with SybrGreen detection and primer sets recommended by the manufacturer (Suppl. Table 3).

### 4.4 Western Blot

Whole cell protein extracts were prepared in RIPA lysis buffer (Sigma) or PhosphoSafe Buffer (Sigma) containing a protease inhibitor cocktail (Roche) and PMSF 10 μg/ml, separated on a 12% SDS-PAGE minigel (Amersham; 50 μg/lane) and transferred to nitrocellulose membranes by electro-blotting. Blots were blocked and developed in Odyssey blocking buffer in 1xPBS/Tween 20. Blots were incubated with primary antibodies overnight (Suppl. Table 4), and β-actin was used as loading control. Secondary antibodies conjugated with IRDye 680 or 800 (1:1000; LI-COR Biosciences, Bad Homburg, Germany) were used for detection. Blots were sequentially developed and analyzed on the Odyssey Infrared Imaging System (LI-COR Biosciences), superimposed and quantified using ImageStudio Light.

### 4.5 Immunoprecipitation

Human embryonic kidney cells (HEK293) were maintained in DMEM medium with 10% FCS and 1% penicillin/streptomycin. The cells were lysed in ice-cold RIPA lysis buffer containing a protease inhibitor cocktail (Roche, Germany) for 45 min. Following centrifugation (12,500 rpm, 45 min, 4 °C) 1 mg of the crude protein lysate was incubated with 20–30 μl A/G PLUS-agarose beads (Santa Cruz Biology, sc-2003) and 5 μl of the respective antibody (anti-NXN, anti-Camk2a) overnight at 4 °C. The beads were centrifuged at 2,500 rpm for 2 min at 4 °C, washed 1x in RIPA lysis buffer, resuspended in 20 μl 4x Laemmli buffer, boiled (10 min, 95 °C) and centrifuged for 2 min at maximum speed (17000 rpm). The supernatant with the immune complexes was separated by 12% SDS-PAGE and transferred to nitrocellulose membranes. Membranes were blocked with 1:1 Odyssey blocking buffer/1x PBS at RT for 1 h. All primary antibody incubations were performed in 1:1 Odyssey blocking buffer/1x PBS-Tween overnight at 4 °C followed by incubation with IRdye-labeled secondary antibodies for 2h at RT. Immunocomplexes were visualized using an Odyssey scanner (LI-COR) according to the manufacturer’s instruction.

### 4.6 Camk2 activity assay

To generate Camk2a enriched protein extracts, the cerebellum was rapidly excised and homogenized on ice in lysis buffer (20 mM Tris HCl pH 8, 137 mM NaCl, 1% Triton X-100, 2 mM EDTA) containing a protease and phosphatase inhibitor cocktails (Sigma). Protein concentrations were assessed with the BCA method. Whole protein extract was incubated with Camk2a antibody (Sigma) for 1h at 4 °C and then with protein C agarose beads (Santa Cruz) overnight at 4 °C. Beads were washed with lysis buffer, pelleted and the supernatant containing Camk2a was subjected to the Camk2 kinase assay (MBL) at a protein content of 200 ng/ml.

The assay was performed in triplicates per condition according to the manufacturer's instructions with Ca^2+^/calmodulin plus kinase buffer (1.25 mM ATP, 125 mM CaCl_2_) to assess total activity, and Ca^2+^/calmodulin minus kinase buffer (1.25 mM ATP, 100 mM EGTA) to measure the tissue-autonomous activity of Camk2. The experiments included a negative control without enzyme, and a positive control with 15 mU Camk2 (MBL). The Camk2a enriched beads were treated with 1 μM recombinant human NXN, 1 μM rNXN plus 1 mM H_2_O_2_, or only 1 mM H_2_O_2_. The absorbance was read at 450 nm on a Tecan Infinite 200Pro ELISA reader, and the percentage of Camk2a activity was calculated relative to the positive control.

### 4.7 Cortical primary neuron culture

Primary cultures of cortical neurons were dissected from postnatal pup brain at postnatal day zero (P0) in 1x PBS (phosphate buffered saline, Gibco) on ice. Cortices were incubated with 1 mg/ml papain (Sigma) activated with l-cysteine at 37 °C for 30 min. After trituration, cells were collected at 4 °C with 800 rpm, and then resuspended in complete Neurobasal medium 2A with 2% (vol/vol) B27 supplement (Gibco), 100 U/ml penicillin/streptomycin, 1% (vol/vol) GlutaMax and plated on poly-d-Lysine coated glass cover slips (5 x 10exp5 cells/ml). Cells were allowed to settle for 2h at 37 °C, and 600 μl maintenance medium (N2A complete, 1 μM Ara-C, 30% astroglial medium) was then added. Half of the medium was exchanged every three days. Cultures were kept at 5% CO_2_, 95% humidity for 14 d until fixation.

Proximity ligation assay and immunofluorescence analysis of primary neurons.

Primary cortical neuron cultures were washed in PBS, fixated in 4% PFA and permeabilized in 0.01% Triton X-100 in 1xPBS. A proximity ligation assay (PLA) was conducted with the Duolink In Situ PLA® kit (Sigma) according to the manufacturer’s protocol. Briefly, cover slips were incubated with blocking buffer for 1h at 37 °C, and then with the primary antibodies directed against NXN and Camk2a at 4 °C overnight. After washing with buffer A, cover slips were incubated with secondary antibodies tagged with PLA probes (anti-mouse PLUS, anti-rabbit MINUS) for 1h at 37 °C in a preheated humidity chamber. After washing in buffer A, the ligase solution was added for 30 min at 37 °C. After washing in buffer A, the amplification was started by adding the polymerase solution, which was topped after 100 min at 37 °C by washing with wash buffer B. For detection of dendritic spines, slides were then incubated overnight at 4 °C with an Alexa488-conjugated antibody directed against microtubule associated protein, MAP2. After final washes with buffer B, slides were mounted in Duolink in situ mounting medium containing DAPI as a nuclear counterstain. Images were captured on the confocal microscope Zeiss LSM 800 (Zeiss, Jena, Germany).

The neighboring localization of fluorescent signals was analyzed in FIJI ImageJ using the Coloc2 colocalization plugin implemented in ImageJ and by overlay analysis of line profile plots along dendrites.

### 4.8 Immunofluorescence and LacZ histology

Mice were terminally anesthetized with carbon dioxide and cardially perfused with cold 0.9% NaCl followed by 2% paraformaldehyde (PFA) for fixation. Tissues were excised, postfixed in 2% PFA for 2 h, cryoprotected overnight in 20% sucrose at 4 °C, embedded in small tissue molds in cryo-medium and cut on a cryotome (10 or 12 μm). Slides were air-dried and stored at −80 °C. After thawing, slides were immersed and permeabilized in 1x PBS with 0.1% Triton-X-100 (PBST), then blocked with 3% BSA/PBST, subsequently incubated overnight with the first primary antibody (Suppl. Table 4) in 1% BSA/PBST at 4 °C. After washing three times with 1x PBS, slides were incubated with the secondary antibodies for 2h at room temperature, followed by 10 min incubation with DAPI and embedding in Aqua-Poly/Mount. The general settings were optimized for the respective antibodies and tissues. Secondary antibodies were labeled with fluorochromes (Invitrogen, Sigma, Life Technologies).

For X-Gal staining, slides of NXN-LacZ reporter mice were washed in PBS and incubated with 4 mg/ml X-Gal (Sigma Aldrich) in dilution buffer (5 mM potassium ferricyanide; 0.01% (w/v) sodium deoxycholate; 2 mM MgCl2; 0.002% (v/v) Nonidet P40) over night at 37 °C. Slides were then washed in PBS, counterstained with eosin for 3 min, washed again in PBS and embedded in Aqua-Poly/Mount.

Microscopic images were captured on an inverted fluorescence microscope (BZ-9000, KEYENCE, Germany or Axio Imager Z1, Zeiss, Germany), or on a Zeiss LSM confocal microscope to assess spatial coexpression of NXN with Camk2a. Tiled images were captured and automatically stitched with Keyene's software or Adobe Phostoshop CC to reconstruct brain regions or spinal cord sections. Subsequently, higher magnification images (20× objective lens) were obtained of regions of interest. Images with analyzed with FIJI ImageJ using background subtraction, threshold settings and plugins implemented in ImageJ. The algorithms included and the particle counter, 2D and 3D intensity plots, and colocalization analysis. Analyses were done with three mice were analyzed per group.

### 4.9 Seahorse analysis of oxygen consumption

Mitochondrial respiration was analyzed with a XFe96 well Seahorse Cell Mito Stress Test (Agilent Technologies) on a Seahorse XFe instrument. It is a 96-well plate-based live cell assay for monitoring of oxygen consumption rates in living cells or protein lysates. Mitochondria were isolated from mouse brain of Nestin-NXN^-/-^ and NXN-flfl mice (n = 3 mice per genotype) using discontinuous Percoll density gradient centrifugation (Qproteome mitochondria, Qiagen). Mitochondrial yield was assessed using a bicinchoninic acid assay of mitochondrial protein. Five μg mitochondria per well (n = 94 for NXN-flfl and n = 69 for Nestin-NXN-/-) were subjected to a “substrate-uncoupler-inhibitor-titration” protocol to assess mitochondrial respiration. After measurement of basal respiration, 4.5 mM ADP plus pyruvate (10 mM)/malate (5 mM) substrate was added to assess ATP generation, followed by oligomycin (3 μM) to observe proton leakage. Subsequently, FCCP (carbonyl cyanide-p-trifluoro-methox-yphenylhydrazone; 4 μM) was added to assess maximum uncoupled respiration. Residual non-mitochondrial respiration was determined after inhibition of complex I with rotenone (2 μM) and complex-III with antimycin A (4 μM). Oxygen consumption (OCR) was analyzed with Seahorse XF Wave® software.

### 4.10 Proteomics

#### 4.10.1 Tissue sampling and protein extraction

Each eight NXN-flfl and Nestin-NXN^-/-^ mice were killed by neck extension, the brain was rapidly removed and tissue pieces of the hippocampus were snap frozen on dry ice and stored in liquid nitrogen pending protein extraction.

Tissue samples were homogenized in 1% Triton X-100, 100 mM Tris pH 7.4 using a motor-driven Potter–Elvehjemn with 15 strokes, and then incubated on ice for 15 min. Following centrifugation at 16000 rpm for 10 min at 4 °C, the supernatant was transferred to a new tube and precipitated with 20% TCA. The pellet was resolved in extraction buffer (10% SDS, 150 mM NaCl, 50 mM HEPES pH 7.8). Precipitated proteins were washed twice with ice-cold acetone and finally resolved in extraction buffer. Sonification for 5 s facilitated resolubilization of proteins. 100 μg of protein from each fraction was diluted in 4% (w/v) SDS, 100 mM HEPES, pH 7.6, 150 mM NaCl, 0.1 M DTT, mixed with 200 μl 8 M urea, 50 mM Tris/HCl, pH 8.5 and loaded onto spin filters with a 30 kDa cut off (Microcon). The filter aided universal sample preparation protocol (FASP) [60] was used as described. Proteins were digested overnight with trypsin/LysC (sequencing grade, Promega). Following the protocol of Kulak and Mann [61], acidified peptides (final concentration 0.1% v/v TCA) were fractionated on multi-stop-and-go tips (StageTips) composed of C18-tips and strong cation exchange (SCX) tips. Peptides from the pellet fraction were eluted in three steps. Peptides from the supernatant fraction were eluted in six steps. All fractions of each sample were eluted in wells of microtiter plates. Peptides were dried and resolved in 1% acetonitrile, 0.1% formic acid.

### 4.11 Biotinylated iodoacetamide (BIAM) switch assay

For the BIAM switch assay, the TCA-aliquot was thawed on ice, centrifuged for 30 min with 16000×*g* and washed with 10% TCA and 5% TCA, respectively. Pellets were resuspended in 200 μl NEM-DAB (8 M urea, 5 mM EDTA, 0.5% SDS, 50 mM Tris/HCl, pH 8.5, 50x molar excess NEM) and incubated at 850 rpm for 1 h at 22 °C in the dark. Proteins were precipitated by ice-cold acetone, collected by centrifugation, washed in acetone, resuspended in 150 μl DTT-DAB (8 M urea, 5 mM EDTA, 0.5% SDS, 50 mM Tris/HCl, pH 8.5, 3 mM DTT) and incubated at 850 rpm for 5 min at 22 °C in the dark followed by addition of 150 μl BIAM-DAB (8 M urea, 5 mM EDTA, 0.5% SDS, 50 mM Tris/HCL, pH 8.5, 50x molar excess BIAM) and incubated for 1 h at 22 °C in the dark. Proteins were precipitated with ice-cold acetone overnight at -20 °C, collected by centrifugation, washed and resuspended in 200 μl lysis buffer (5 mM EDTA, 50 mM Tris/HCl pH 8.5, 1% Triton-X-100, 1% SDS). 200 μg of proteins were affinity purified using agarose streptavidin beads overnight at 4 °C on a wheel. The supernatant of the affinity purification was digested for further analysis of overoxidized proteins. After washing, beads were resuspended in 50 μl 6 M GdmCl, 50 mM Tris/HCl, pH 8.5 and incubated at 95 °C for 5 min. Sample were diluted with 25 mM Tris/HCl, pH 8.5, 10% acetonitrile to obtain a final GdmCl concentration of 0.6 M. Proteins were digested with 1 μg Trypsin overnight at 37 °C under gentle agitation. Digestion was stopped by adding TCA to a final concentration of 0.5%. Peptides were loaded on multi-stop-and-go tip (StageTip) containing three C18 discs and three SCX discs. Peptides were eluted in wells of microtiter plates and peptides were dried and resolved in 1% acetonitrile, 0.1% formic acid.

### 4.12 Label free mass spectrometry

For proteome analysis, the LC/MS was performed on a Thermo Scientific™ Q Exactive Plus equipped with an ultra-high-performance liquid chromatography unit (Thermo Scientific Dionex Ultimate 3000) and a Nanospray Flex Ion-Source (Thermo Scientific). Peptides were loaded on a C18 reversed-phase pre-column (Thermo Scientific) followed by separation on in-house packed picotip emitter tips (diameter 100 μm, 15 cm long from New Objectives) with 2.4 μm Reprosil C18 resin (Dr. Maisch GmbH). The gradient was from mobile phase A (4% acetonitrile, 0.1% formic acid) to 80% mobile phase B (80% acetonitrile, 0.1% formic acid) for 90 min with a flow rate 400 nl/min.

MS data were recorded by data dependent acquisition Top10 method selecting the most abundant precursor ions in positive mode for HCD fragmentation. Lock mass option [62] was enabled to ensure high mass accuracy between multiple runs. The full MS scan range was 300 to 2000 *m/z* with resolution of 70000 and an automatic gain control (AGC) value of 3*10^6^ total ion counts. The maximal ion injection time was 160 ms. Higher charged ions (2+) were selected for MS/MS scans with a resolution of 17500, an isolation window of 2 *m/z* and an automatic gain control value set to 10^5^ ions. Ions were excluded if they occurred within a time-window of 20 s following a fragmentation event. Full scan data were acquired in profile mode and fragments in centroid mode by Xcalibur software.

### 4.13 Proteomic data analyses

For data analysis MaxQuant 1.6.1.0, Perseus 1.6.1.5 and ArrayStar (DNASTAR 18) were used. N-terminal acetylation (+42.01) and oxidation of methionine (+15.99), N-ethylmaleimide on cysteines (+125.05) and biotinylated iodoacetamide (+414.19) were selected as variable modifications for BIAM-Switch. The mouse reference proteome set (Uniprot, July 2020) was used as template to identify peptides and proteins with a false discovery rate (FDR) less than 1%. The minimal ratio count for label-free quantification (LFQ) was 1. The mass spectrometry proteomics data have been deposited to the ProteomeXchange Consortium via the PRIDE [26] partner repository with the dataset identifier PXD024624. reviewer_pxd024624@ebi.ac.uk.

Proteins were quality filtered according to unique peptides, sequence coverage, putative contaminants and a minimum of 8 valid values in total out of 32 samples. LFQ protein intensities were Log2 transformed and single missing/zero values in the full proteome were imputed from the normal distribution. Differences between genotypes for full proteome and BIAM proteome were assessed with t-tests and plotted as Volcano plots where the Log2 difference (= fold difference) is plotted on the X-axis versus the negative Log10 value of the P-value on the Y-axis. Hierarchical clustering (Ward method) was employed to further assess protein patterns and genotype differences using Euclidean (squared) distance metrics. Regulated proteins (1.5fold, 90% confidence) were submitted to the "functional gene clustering" tools of The Database for Annotation, Visualization and Integrated Discovery (DAVID, http://david.abcc.ncifcrf.gov), and networks were assessed in STRING (https://string-db.org/). The P value was set at 0.05 adjusted according to Benjamini Hochberg.

### 4.14 Behavioral analyses

Behavioral analyses were done with unbiased video-based or antenna based automated observation and analysis. Groups were matched according to age and gender, and littermates were used as far as possible. Most experiments were done with male and female mice except in IntelliCages where only females were used to avoid social fights. A summary of sample sizes and results is in Suppl. Table 2.

### 4.15 Phenomaster

The TSE Phenomaster provides automated high precision monitoring of feeding, drinking and voluntary wheel running (VWR) in a home cage environment. Drinking and feeding behavior were monitored with high-precision weight sensors for liquid and food dispensers, which are integrated into the lid of the cage. The running wheel was freely accessible and braking adjusted to allow for appetitive running. Mice were adapted to the drinking bottles for one week in their home cage and to the Phenomaster cage for one day before starting the experiment. Drinking, feeding and voluntary wheel running were recorded for 24 h.

### 4.16 Open Field (OFT) and Elevated Plus Maze (EPM)

Mice were placed in the middle of an Open Field (50 X 50 cm width, 38 cm height) and allowed to move freely for 10 min. They were observed per video camera. Virtual zones were defined as centre and border.

In the EPM test, mice were placed in the centre of a standard EPM with 2 open arms and 2 closed arms with grey plastic walls (10 x 50 cm, height 50 cm above ground) and allowed to move freely for 10 min.

In both tests, locomotion, visits to and times spent in zones were analyzed with VideoMot2 which uses 2-point tracking (TSE Systems).

### 4.17 Social cognition & memory

The social discrimination task assesses social cognition and memory according to standard protocols in a three-chamber box (each chamber 18.5 x 38 cm) [63]. The middle chamber was connected to the outer chambers by doors, which can be closed. A cylindrical enclosure was placed into the corners of each outer compartment. Mice were habituated to the environment before start. At the experiment day, mice were acclimatized to the middle chamber for 5 min with closed doors. The doors were then opened and mice allowed to explore the chambers and enclosures, one empty, the other with a stimulus mouse for 10 min (social cognition). Subsequently, a second mouse was added to the still empty enclosure, again for 10 min to assess behavior towards social novelty. The trials were recorded with a video camera and analyzed with VideoMot2 software (TSE Systems).

### 4.18 Touchscreen cognitive performance

Bussey–Saksida touchscreen equipment (Campden Instruments Ltd/Lafayette Instruments) was used as described [64]. The trapezoidal operant chamber consists of an infrared touchscreen spanning the wider end of the trapezoid, a perforated ﬂoor and a peristaltic liquid reward supply at the narrow end of the trapezoid. Presentation of stimuli, nosepokes on the screen and capture of the behavior with video cameras were controlled by Lafayette ABET II software. The touchscreen trainings and tasks are summarized in Suppl. Table 5.

Touch screen – 5-choice serial reaction time (5-CSRT)

One week prior to the habituation phase, mice were set on a restriction diet with 2–3 g of food pellets per day to reduce the body weight to 90%. The diet was maintained throughout experiments to increase the appetite for the liquid reward, which consisted in 8 μl sweetened condensed milk (Nestlé, Switzerland; diluted 1:4 in tap water). For stimulus presentation within the 5CSRT task, a black Perspex mask with five windows was placed in front of the touchscreen. The training steps were adapted from the procedures published by the Bussey and Holmes laboratories [65,66]. During the training stages, the animals learned to touch the screen in correct locations to get a tone-coupled reward. In the “Must-Touch” pre-training, the mouse had to touch the screen at any site in response to the stimulus to collect the reward. In the next “Must Initiate” training, the mouse had to trigger the stimulus by poking into the illuminated reward through. The trigger-to-stimulus delay was 5 s. The criterion of success was deﬁned as completion of 30 trials within 60 min on two consecutive training days. For the 5CSRT task, a white-square is presented pseudo-randomly in one out of five possible locations, and the mouse has to touch the screen in the correct position. Outside of the lit rectangle, the screen is dark. In the “Punish Incorrect” training, settings were as in Must Initiate but a timeout period of 5 s was triggered if the mouse touched the blank site of the screen outside of the enlightened square. During timeout, the image disappeared and the overhead lighting (~60 Lux) was turned on for 5 s. The criterion was to complete 30 trials in 60 min with >75% correct responses. During the 5CSRT testing phase, settings remained identical to Punish Incorrect, but the time of stimulus presentation was progressively decreased (32 s, 16 s, 8 s, 4 s and 2 s). For each stimulus time, mice performed three sessions (one session per day). The 5CSRT task primarily addresses attention, responses to short visual stimuli, spatial discrimination and impulse control. Sustained attention and translation into action in the 5CSRT depends on dopaminergic neurons in Nc. accumbens [67] and GABAergic systems in medial prefrontal cortex [68].

### 4.19 Touch screen - pairwise discrimination (PD)

The Pairwise Discrimination task was done after completion of the 5CSRT. For stimulus presentation, a black perspex mask with two windows was placed in front of the touch screen. During training stages, a white simple pictogram of an object on black background (Campden/Lafayette software) was presented in one of the two windows. The mice had to touch the pictogram-window in order to elicit a tone-coupled reward. During test sessions, two novel stimuli (pictogram) were presented in a spatially pseudo randomized order over 30-trial sessions (20 s intertrial-interval, ITI) in the absence of overhead lighting. One image was set to correct, the other to incorrect. Responses to the correct stimulus resulted in 8 μl reward. Responses to the incorrect stimulus resulted in a 5 s timeout, coupled with switching the ~60 lux house light on. This was followed by a correction trial. Stimuli remained on the screen until a response was made [[69], [70], [71]]. The criterion to enter the reversal stage was to complete 30 trials in 60 min with >75% correct responses, for a minimum of 3 consecutive testing days. During the reversal stage, the previously correct image was set to incorrect and coupled with overhead lighting for 5 s. Inversely the previously unrewarded image was set to correct and elicited the tone-coupled reward supply. The criterion for reversal learning was an average percent correctness of 80% or higher. The performance in the PD task involves functioning of glutamatergic and muscarinic systems [[71], [72], [73]].

### 4.20 IntelliCage

The IntelliCage (NewBehavior AG, Zurich, Switzerland) [30,51,[74], [75], [76]] consists of four operant corners, each with two water bottles, sensors, light-emitting-diodes (LEDs) and doors that control the access to the water bottles. The system ﬁts into a large cage (20 × 55 × 38 cm, Tecniplast, 2000P) and allows housing of 16 mice per cage. Four triangular red shelters (Tecniplast) are placed in the center to serve as sleeping quarters and as stands to reach the food. The ﬂoor is covered with standard bedding. Mice are tagged with radio-frequency identiﬁcation (RFID)-transponders, which are read with an RFID antenna integrated at corner entrance. Inside the corners, there are two holes with water bottles, which can be opened and closed by automated doors. Mice have to make nosepokes (NP) to open the doors for water access. The IntelliCage is controlled by a computer with IntelliCage Plus software, which executes pre-programmed experimental tasks and schedules. The numbers and duration of corner visits, nosepokes, and licks are automatically recorded without the need for handling of the mice during the recording times.

### 4.21 IntelliCage - behavioral tasks

IntelliCage tasks address a number of different aspects of cognition as well as circadian rhythms and social interactions, and were run sequentially. The tasks are described in Suppl. Table 6, and abbreviations of behavioral parameters are summarized in Suppl. Table 7. The tasks followed established protocols [30,45,51]. The IntelliCage experiments were done in female mice to avoid fighting. Up to 16 mice were housed per cage (8/8 and 7/8 of each genotype). Mice in cage-1 were 5–6 months old upon start, mice in cage-2 were 11–12 months.

Mice were adapted to the system for 8 days with free access to every corner, with all doors open, and water and food ad libitum. This free adaptation (FA) was followed by 6-days nosepoke adaptation (NP) during which the doors were closed, the ﬁrst nosepoke of the visit opened the door for 5 s and in order to drink more, the animals had to leave the corner and start a new visit. In the place preference learning (PPL) task mice had to learn to prefer a specific corner for 8 days, where they got the water reward. Each 4 mice were assigned to one corner and the PPL module was active for 24h. Only the first correct nosepoke opened the door, and an incorrect nosepokes had no effect. After conditioning to the corner, PPL reversal learning (PPLrev) was assessed by switching the rewarding corner to the opposite side for 7 days, and another corner switch for 13 days (PPLrev2). Subsequently, LED attention and visual discrimination were assessed in an NP protocol for 7 days, where an LED was randomly switched on, either on the right or left side upon corner entry, and only the LED side was set to correct. Salience based motivation has been assigned to cortical regions and the nucleus accumbens [77,78]. Flexible spatial Reversal learning requires the dorsal and ventral hippocampus and their functional interactions with the prefrontal cortex [79,80], and is partially lost with disturbances of adult hippocampal neurogenesis [81].

In place avoidance learning (PAL), mice had to learn to avoid one punished corner, which was randomly assigned to each 4 mice. The punishment consisted in an airpuff (~ 0.8 bar, 1 s) and was coupled with red LED. Avoidance acquisition lasted for 24h. At completion, mice returned to their home cages for 1 day with water restriction for the last 18 h prior to their return to their IntelliCage for the analysis of the extinction of the avoidance behavior for 6 days (PAEx). The temporary water restriction ensured that all mice were equally thirsty and highly motivated to get water upon re-entry of the IntelliCage. The IntelliCage was not cleaned during the home cage stay to maintain the environmental and olfactory cues. In PAEx, all doors opened in response to a nosepoke and no air-puff was applied. Only the red LED still reminded of the previously 'punished' corner. Conditioned place avoidance in the IntelliCage is sensitive to functions of the hippocampus [75] and reminiscent of fear conditioning in classical foot-shock based tests of hippocampal functions.

Finally, the last NP protocol was run with tap water on one side and sweet water on the other side of each corner to assess the sugar appeal (7 days).

## 5 Statistics

Group data are presented as mean ± SD or median ± IQR for non-parametric data as specified in the respective figure legends. Data were analyzed with SPSS 24 and Graphpad Prism 8.4 or 9.0 and Origin Pro 2021. Data were mostly normally distributed, or log-normally distributed. For testing the null-hypothesis that groups were identical, two groups were compared with 2-sided, unpaired Student's t-tests. The Mann Whitney *U* test (2 groups) was used as a non-parametric alternative in case of violations of *t*-test requirements. Time course data or multifactorial data were submitted to 2-way analysis of variance (ANOVA) using e.g. the factors 'time' and 'genotype'. In case of significant differences, groups were mutually compared at individual time points using post hoc t-tests according to Dunnett, i.e. versus the control group, or according to Šidák. The FDR was used for proteomic data. Asterisks in figures show multiplicity-adjusted P-values.

Multivariate behavioral parameters were used to reduce the dimensionality. To assess different behavioral readouts together, data were normalized and are expressed as percentage of the 90%-quantile. Canonical discriminant analyses (CanDisc) was employed to separate treatment groups and assess the predictability of group membership. Polar plots were created in Origin Pro 2021. Chi-square automatic interaction detection (CHAID) was used to generate decision trees, which is based on Bonferroni adjusted significance testing. Behavioral parameters were introduced as independent variables, and then stepwise removed to find the strongest discriminant candidates.

IntelliCage data were analyzed with IntelliCage Plus® software (TSE) and with FlowR (NewBehavior) [76,82] for analyses of time courses and circadian parameters. Data were exported to tab separated txt files for further analyses in Excel, Prism, SPSS and OriginPro. The day-mean behavior for each module for each mouse was used for 2-way ANOVA for “module” by “genotype”. The mean behavior per mouse for each parameter over the total observation time was used for comparisons of box plots and distributions.

## Data availability statement

The mass spectrometry proteomics data have been deposited to the ProteomeXchange Consortium via the PRIDE [26] partner repository with the dataset identifier PXD024624.

## Author contributions

BT and LV performed the behavioral analyses, in vitro Camk2 assay, immunohistochemistry, primary culture, imaging, and analyzed data. AWS maintained the mouse colonies and prepared tissue. IW did the proteomics, DCF and DN provided experimental help for Seahorse experiments, IT initiated the study, organized and designed the experiments, coordinated sub-projects, analyzed data, created the figures and wrote the manuscript. All authors contributed to writing or revising the manuscript and approved the final version of the manuscript.

## Declaration of competing interest

The authors declare that they have no competing financial interests or other competing interests that might be perceived to influence the results and/or discussion reported in this paper.
