# Supplementary material for: Reduced exploratory behavior in neuronal nucleoredoxin knockout mice
Source: Redox Biol. 2021 Jun 23;45:102054. doi: 10.1016/j.redox.2021.102054 (PMC8254043; doi:10.1016/j.redox.2021.102054)
Supplement: Multimedia component 1 [file mmc1.docx]

# Supplementary Tables

## Suppl. Table 1

### Yeast-2-hybrid report summary

##

Abbreviations and links are provided in the accompanying supplementary Excel file

## Suppl. Table 2

### Behavioral experiments: Overview of gender, ages, experiments

| Experiment | Readout | Interpretation | Gender and age NXN-flfl | Gender and age NesNxn-/- | Genotype effect | Results  of NesNXN-/- |
| --- | --- | --- | --- | --- | --- | --- |
| Body weight | Body weight time course | Basic health | 17 f (9-12 wks); 10 m (8-12 wks) | 20 f (8-15 wks); 9 m (8-12 wks) | YES | Lower body weight of female young |
| Phenomaster | Feeding & drinking | Basic health, sucrose appeal | 6 f (8-9 wks); 5 m (11 wks) | 6 f (8-9 wks); 5 m (11-15 wks) | NO | Equivalent feeding & drinking |
| Phenomaster | Voluntary wheel running | VWR hedonia | 6 f (8-9 wks); 5 m (11 wks) | 6 f (8-9 wks); 5 m (11-15 wks) | YES | Reduced VWR |
| Open field (OFT) | Travel paths, times in zones | Exploration, locomotion | 7 f (8-12 wks) + 8 m (9-12 wks) | 10 f (10-15 wks) + 4 m (9-11 wks) | NO | Reduced center exploration |
| Elevated Plus Maze (EPM) | Time in Open, Closed | Anxiety, curiosity | 7 f (8-12 wks) + 8 m (9-12 wks) | 10 f (10-15 wks) + 4 m (9-11 wks) | NO | Equivalent time in Open and Closed |
| Sociability | Time with object/social | Social cognition & memory | 7 f (8-12 wks) | 7 f (8-12 wks) | NO | Equivalent paths and preferences |
| Touchscreen | 5-Choice Serial Reaction Task (5CSRT) | Attention & Learning | 5 f (9-12 wks); 3 m (8-12 wks) | 5 f (8-12 wks); 2 m (12 wks) | NO | Equivalent latencies and accuracy |
| Touchscreen | Pairwise Discrimination Task (2CDT) | Learning & Visual discrimination | 4 f (21-24 wks); 4 m (20-24 wks) | 6 f (18-24 wks); 2 m (21 wks) | NO | Equivalent latencies and accuracy |
| Intellicage | Activities (Visits, Nosepokes Licking) | Activity, exploration, circadian, social | 8 f (31-44 wks); 8 f (27-28 wks) | 7 f (39-44 wks); 8 f (27-29 wks) | YES | Reduced exploratory behavior |
| Intellicage | Learning & Memory | Cognitive functions | 8 f (31-44 wks); 8 f (27-28 wks) | 7 f (39-44 wks); 8 f (27-29 wks) | YES | Faster preference learning |

## Suppl. Table 3: Genotyping primer and PCR primer (5’ > 3’)

| Name | Sequence | Allele | Length |
| --- | --- | --- | --- |
| CAS_R1_Term | TCGTGGTATCGTTATGCGCC | NXN floxed allele | 204 |
| Nxn_345_F | TTCCCAGGTTCTCAGCTTCC | NXN wildtype allele | 345 |
| Nxn_345_R | CATCTTGGAGTGCAATGAGACC | NXN wildtype allele | 345 |
| LacZ_2_small_F | ATCACGACGCGCTGTATC L | LacZ | 108 |
| LacZ_2_small_R | ACATCGGGCAAATAATATCG | LacZ | 108 |
| CRE Forward | GAAAGCAGCCATGTCCAATTTACTGACCGTAC | Cre |  |
| CRE Reverse | GCGCGCCTGAAGATATAGAAGA | Cre |  |
| rtPCR Primer |  |  |  |
| Mouse NXN | GGAGGTGCTCAATGACGAGGA  GGAGGTGCTCAATGACGAGGA | Nxn |  |
| PP1A | GCTGGACCAAACACAAACGG  GCCATTCCTGGACCCAAA AC | Housekeeper PP1A |  |

## Suppl. Table 4: Antibodies

| Antibody | Manufacturer | Product # | Dilution | Host | Type |
| --- | --- | --- | --- | --- | --- |
| NXN | Sigma | HPA023566 | 1:200 | rabbit | pab |
| Camk2a | Invitrogen | MA1-048 | 1:500 | mouse | mab |
| Camk2a | Abcam | ab22609 | 1:50 (IP) | mouse | mab |
| β-actin | Sigma | A5441 | 1:1000 | mouse | mab |
| GAPDH | Ambion | AM4300 | 1:1000 | mouse | mab |
| NeuN | Millipore | MAB377 | 1:200 | mouse | mab |
| GFAP | Millipore | MAB 360 | 1:200 | mouse | mab |
| MAP2-Alexa488 | Sigma | MAB3418X | 1:400 | mouse | mab |

## Suppl. Table 5 Touchscreen trainings

| # | Touchscreen Experiment | Touchscreen task name | Description | Sessions | Criterion for success |
| --- | --- | --- | --- | --- | --- |
| 0 | 5CSRT and PD | Motivation diet | Food pellets are reduced to 2-3 g per mouse/day to reduce and keep bodyweight at 90% of baseline. In paralle mice are habituated to sweetened condensed milk (1:4 in tap water) | during th whole experiment | 10% reduction diet (for adult, not growing mice) |
| 1 | 5CSRT and PD | Cage Habituation | Mice are set in TS cage for 40 min and receive 150 µl sweetened condensed milk | 1-2 days | Mice must drink the offered sweetened condensed milk completely to reach the next phase |
| 2 | 5CSRT | Habituation 2v2 | Mice start with 150 µl sweetened condensed milk (prime), then mice receive 8 µl sweetened condensed milk every 10 s along with an auditory signal to associate the signal tone with the reward | 2 days | Mice must complete 30 trials during the session ((1st session with 40 min, 2nd session with 60 min) for 2 consecutive days |
| 3 | 5CSRT and PD | Must Touch | The screen displays an image of five rectangles, and mice have to touch it (nosepoke) in order to receive reward coupled to the signaling tone | 2-7 days | Mice must complete 30 correct trials (touching the image on the screen) during the session ((1st session with 40 min, 2nd session with 60 min) for 2 consecutive days |
| 4 | 5CSRT and PD | Must Initiate Touch | Mice must initiate the image display by putting the head into the food tray, then make a nosepoke on the screen to receive their reward. | 2 | Mice must complete 30 correct trials (touching the picture on the screen) and initiate the next image display during the session (1st session with 40 min, 2nd 60 min) for 2 consecutive days |
| 5 | 5CSRT and PD | Punish Incorrrect | As Must Initiate Touch, mice have to initiate the next display of the picture by putting their head into the food tray. But a nosepoke on the screen outside the image is considered as a failed trial. In consequence the house light goes on along with a time out of 5 s and 5 s ITI (inter trial interval), and the picture disappears. For **5CSRT:** A white square appears in 1 out of 5 possible positions. For **Pairwise Discrimination** (PD): an image (a random picture from a selection of 40 images) is presented in one of the two positions | 3-18 days | Mice must complete 30 trials during one session (60 min) with a correctness of >60-75% (depends on age) for 3 consecutive days. A correct trial is a nosepoke on the screen in the correct position. |
| 6 | 5CSRT: 5-Choice Serial Reaction Time Task | 5CSRT Basic v3 | Same as in Punish Incorrect, but the display time of the picture (Stimulus duration) is restricted to 32, 16, 8, 4 or 2 s. If the mouse does not react to the stimulus (omission) or performs an incorrect touch, there will be a punishment (timeout) as in Punish Incorrect. | 3 per stimulus time | Mice must complete 40-60 trials during one session (60 min) with a correctness of >60-75% depending on age and an omission rate <20% for 2 consecutive days. If >75% of mice fail, the experiment is finished. |
| 7 | PD: Discrimination of pairwise presented easy pictograms such as flower and airoplane | Pairwise Discrimination v3 (Initial) | Mice learn to discriminate between two novel images. One is randomly designated as "correct". The images are presented on right or left side pseudo-randomized. The Intertrial INterval (ITI) is 15 s. Correct responses are rewarded. Each incorrect response is followed by a correction trial, in which the images is presented as in the previous trial, until a correct response is made. | 6 days | Mice must complete 30 trials during one session (60 min) with a correctness of >60-75% depending on age for 3 consecutive days |
| 8 | PD REVERSAL | Pairwise Discrimination v3 (Reversal) | Settings like in PD Intial, but the designation of correct and incorrect images is switched, i.e. the previously rewarded picture is now incorrect and vice versa | 8 days | Mice must complete 30 trials during one session (60 min) with correctness of >60-75 % depending on age for 3 consecutive days |

## Suppl. Table 6 IntelliCage Tasks

| Task name | Task description | Duration | Readouts, cognitive dimensions and interpretation |
| --- | --- | --- | --- |
| Free adaptation (FA) | Habituation to the system with free access to every corner, with all doors open, and water and food ad libitum. | 8 days | Exploratory behavior, general activity, circadian rhythms, social structure |
| Nosepoke adaptation (NP) | The first nosepoke of a visit opened the door for 5 s. To drink more, the animals had to leave the corner and start a new visit. | 6 days | Exploratory behavior, general activity, circadian rhythms, social structure |
| Place preference learning (PPL) | Mice were allowed to drink in one out of 4 corners. Only the first correct nosepoke of a visit opened the door. Three-color LEDs announced entry to correct corner. Each 4 mice assigned to one correct corner | 8 days | Simple spatial preference learning activity and exploration |
| Place preference REVERSAL (PPLrev) | Protocol as in “place preference learning” but with the opposite corner being correct. | 7 days | Cognitive flexibility of Reversal Learning requires the dorsal and ventral hippocampus and their functional interactions with the prefrontal cortex [2, 3] |
| Place preference REVERSAL 2 (PPLrev2) | Protocol as in “place preference learning” but new assignment of correct corner and only one side in this corner was correct. Each 5-6 mice assigned to one correct corner. One corner was excluded | 13 days | Reversal Learning plus only one side out of 8 sides correct. |
| NP random side (NPrd) | Upon corner entry, one side was randomly assigned to be correct, which was indicated by LED. NP opened the door on this correct side. The other side remained closed. | 7 days | Requires attention to LED and decision for correct side according to LED. Requires functions of the hippocampus and medial PFC [5, 6] |
| Place avoidance acquisition (PAA) | Mice had to avoid one specific previously preferred corner. NP in this corner was punished with an air-puff and a red LED was switched on upon visit to this corner. Avoidance acquisition was for 24h, followed by a one-day home cage interval. Each 4 mice assigned to one forbidden corner | 1 day | Spatial avoidance learning is sensitive to genetic differences and hippocampal lesions [7] |
| Place avoidance extinction (PAEx) | During avoidance extinction, water was available in each corner on NP without any punishment. Only the red LED still announced the previously punished corner. | 6 days | Retention of avoidance behavior. Avoidance memory. Duration of avoidance reflects caution over curiosity |
| Final free adapation (FAfin) with one sweet corner | Final free access protocol with all doors open, and water and food ad libitum. In one corner sweet water was provided. | 7 days | Exploratory behavior, sweet preference, general activity, circadian rhythms, social structure |

## Suppl. Table 7

### Abbreviations of behavioral parameters of IntelliCage experiments

| Visits | Visits / h |
| --- | --- |
| NPvisits | Visits with Nosepoke without Licks / h |
| Lvisits | Visits with Licks / h |
| SVisits | Visits without Licks and without Nosepokes / h |
| NPVdur | Median duration of Visits with NP w/out Lick (s) |
| Nosepokes (NP) | Mean number of Nosepokes during Visits with NP w/out Licks |
| NPduration | Median duration of such Nosepokes during a Visit (s) |
| Licks | Median number of Licks per Visit |
| Lduration | Median duration of Licking during a Visit (s) |
| Lcontact | Median bottle cap contact time during a Visit (s) |
| Nocturnal | Log(Visit frequency during dark phase / Visit frequency during light phase) |
| Repetitive | Repetitiveness, log(sum of observed returns to same corner / sum of expected such switches) |
| Regularity | Sqrt (sum of sq non-diag. transition matrix residuals / sum of non-diag. transition matrix observed values) |
| IVI | Intervisit intervals (s) i.e. time from end of visit to start of next corner visit |
| IVIrepdens | Intervisit intervals (s) for repeated use of the same corner |
| InstantFreq | Instantaneous frequency, reciprocal of the time from start of one visit to start of next visit |
| Unevenness | Describes the relative use of corners, ranges from 0-1 (0=equal use of 4 corners, 1=exclusive use of 1 corner) |
| Sidedness | Ratio of visits with first left versus first right NP of visits with NPs |
| Mesor | **M**idline **e**stimating **s**tatistic **o**f **r**hythm. The mesor is a circadian rhythm-adjusted mean based on the parameters of a cosine function fitted to the raw data of the visits. |
| Amplitude | Difference between Mesor and Peak activity |
| Acrophase | Time to maximum activity after Light Off (Light off set to 0) |
| Period | Duration of one circadian cycle |
