# Supplementary material for: Reduced exploratory behavior in neuronal nucleoredoxin knockout mice
Source: Redox Biol. 2021 Jun 23;45:102054. doi: 10.1016/j.redox.2021.102054 (PMC8254043; doi:10.1016/j.redox.2021.102054)
Supplement: Multimedia component 3 [file mmc3.docx]

Supplementary figures and legends to:

Reduced exploratory behavior in neuronal nucleoredoxin knockout mice

#
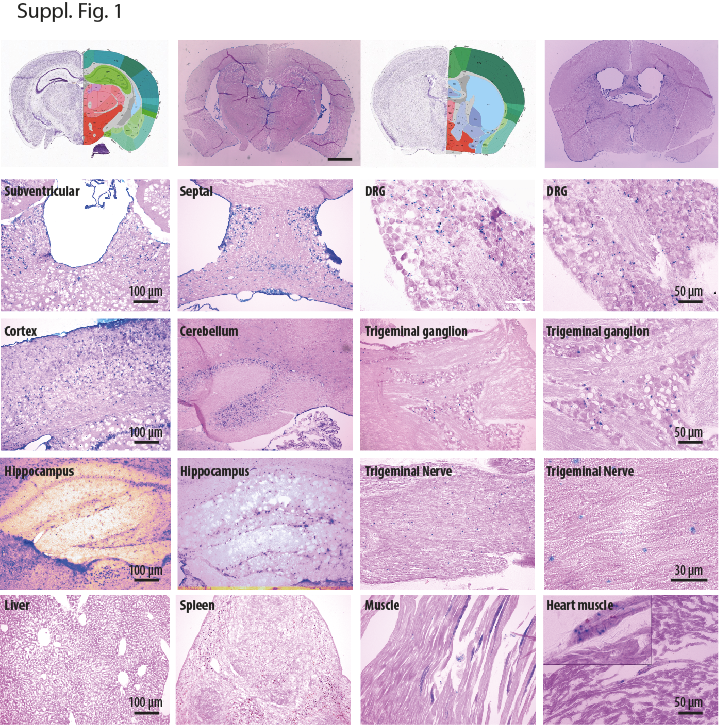


# Suppl. Figure 1

## Beta galactosidase histology of NXN-LacZ mice

Cryosections of adult NXN-LacZ reporter mice were stained with X-gal, followed by eosin counterstaining. The top images show overviews and the respective region maps from the mouse Brain Atlas. Strong expression in nervous tissue included septal nuclei, hippocampus, cortex, cerebellum and sensory ganglia. The bottom row shows LacZ in skeletal muscle and heart muscle but not in the liver.

#
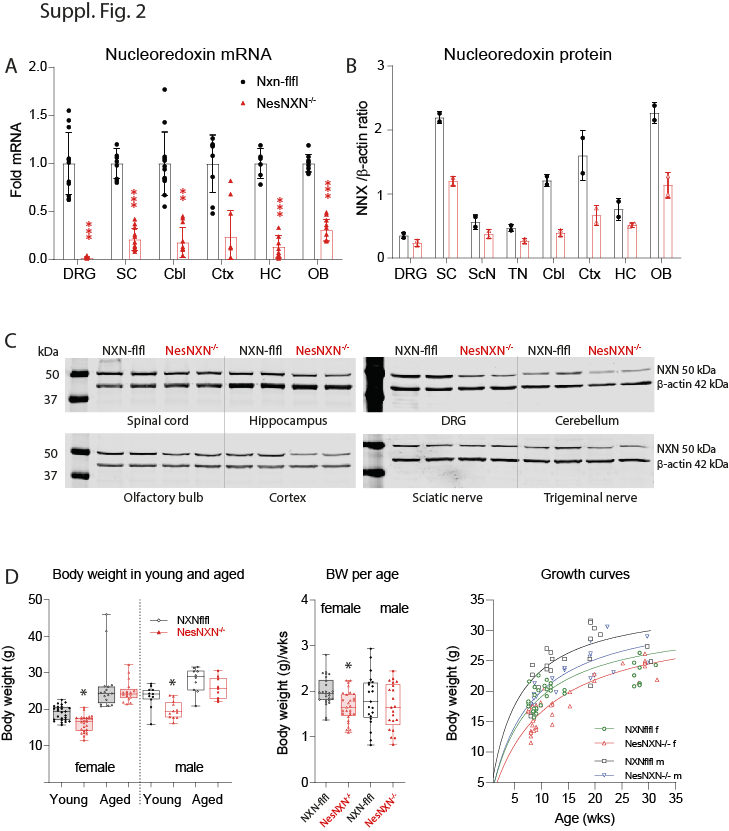


# Suppl. Figure 2

## Nucleoredoxin expression and basic health of Nestin-NXN^-/-^ mice

Nestin-NXN^-/-^ mice were generated by breeding Nestin-Cre with NXN-flfl mice.

**A:** QRT-PCR analysis of NXN in different regions of the central and peripheral nervous system. NXN RNA was reduced by about 80-90 % in Nestin-NXN^-/-^ mice as compared to NXN-flfl mice.

**B, C:** Western Blot analysis of NXN expression in different brain regions in Nestin-NXN^-/-^ and NXN-flfl mice. B shows the quantification and C shows Western Blot examples. NXN protein expression was decreased by about 50%.

**D:** Body weights of Nestin-NXN^-/-^ and NXN-flfl mice at different ages. Young mice were 7-9 weeks and aged mice 25-50 weeks old. Growth curves were fitted to an exponential increase. Each scatter represents a mouse. Data were compared with 2-way or one-way ANOVA and subsequent posthoc analysis using an adjustment of alpha according to Šidák. Asterisks show significant differences between genotype, *P<0.05.

#
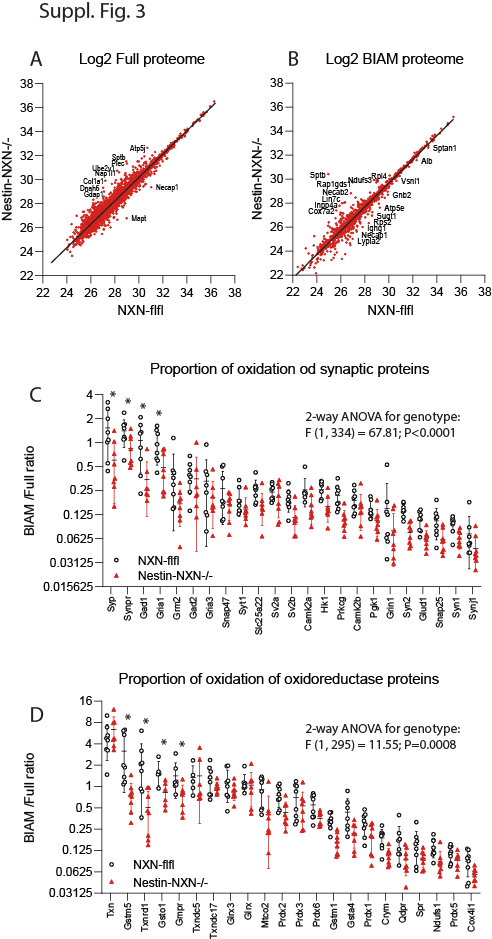


# Suppl. Figure 3

## Scatter plots of protein expression and oxidation in the hippocampus

**A, B:** Scatter plots of protein expression (full proteome) and protein oxidation (BIAM proteome) in the hippocampus of adult NXN-flfl mice (x-axis) versus Nestin-NXN-/- (Y-axis). The plots show the Log2-label free quantification (LFQ) and the linear regression line. Proteins or oxidized proteins above the regression line were increased in Nestin-NXN-/- mice, proteins below were reduced.

**C, D:** Scatter plots of the proportion of protein oxidation (BIAM/Full proteome ratio) of proteins associated with the GO-term "synapse" and "oxidoreduction processes". These GO terms (and "mitochondrion" in Figure 3, main body) were significantly regulated in Nestin-NXN-/- mice versus NXN-flfl mice. The proportion of protein oxidation was reduced in Nestin-NXN-/- mice. The asterisks show significant differences of individual proteins, P value <0.05 adjusted according to Benjamini-Hochberg FDR.

#

#
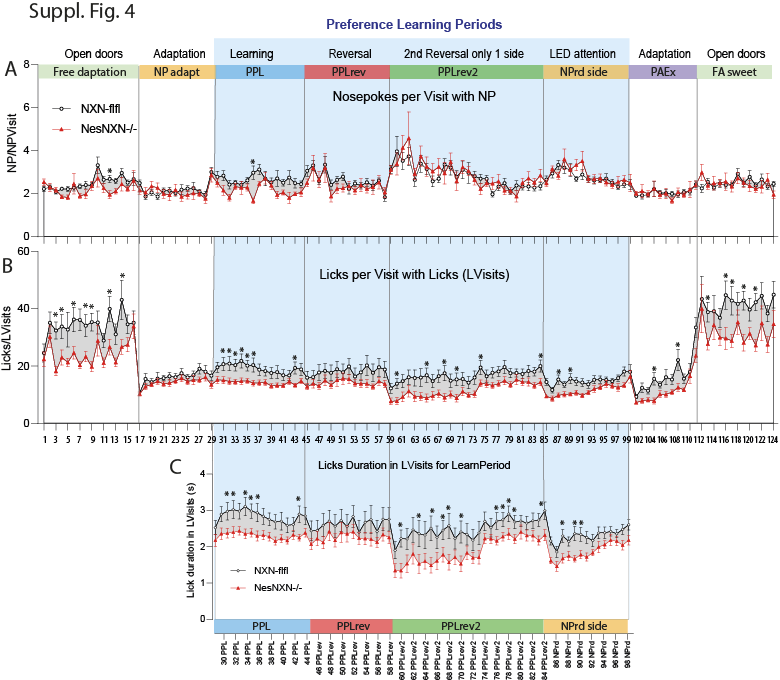


# Suppl. Figure 4

## Reward collecting behavior of Nestin-NXN^-/-^ versus NXN-flfl mice in IntelliCages

**A:** Time courses of nosepokes per visit for visits with nosepokes (NP/NPvisit) and

**B:** Time course of the number of licks per visit with licks (Licks/LVisit) during different tasks in IntelliCages (tasks in Suppl. Table 6, abbreviations Suppl. Table 7). The data show means ± sem of 14-15 mice per group. The fluctuation of the behavior reveals nighttime and daytime differences (12h Bins). The number of licks per visit shows reward collection for successful goal-directed LVisits.

**C:** Mean duration of licking during successful LVisits during the place preference learning periods.
Data were compared with 2-way ANOVA for the factors “time” X “genotype” and posthoc comparison for “genotype” with adjustment of alpha according to Šidák.

#
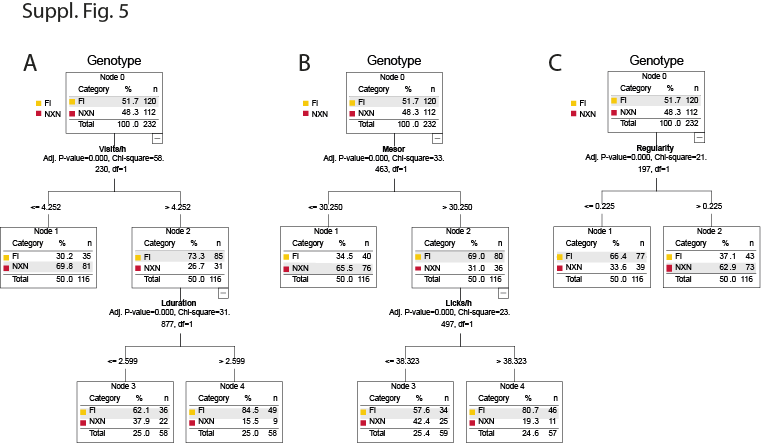


# Suppl. Figure 5

## Decision trees of CHAID analysis of multimodal behavior

**A, B, C:** Behavioral data were normalized on the median of the NXN-flfl control mice and then submitted to CHAID ((Chi-square automatic interaction detection) analysis to find the behavioral parameters which differed most between groups and were able to separate groups. The analysis started with all parameters presented in Figure 7 (polar plots of multimodal behavior) and then stepwise removed the top separators. Hence, in A the decision was based on Visits/h, this was then removed from the input, showing Mesor in B as next candidate, and subsequently the regularity in C.
